# Evaluation of Pseudopteroxazole and Pseudopterosin Derivatives against *Mycobacterium* *tuberculosis* and Other Pathogens

**DOI:** 10.3390/md10081711

**Published:** 2012-08-15

**Authors:** Malcolm W. B. McCulloch, Brad Haltli, Douglas H. Marchbank, Russell G. Kerr

**Affiliations:** 1 Department of Chemistry and Department of Biomedical Sciences, Atlantic Veterinary College, University of Prince Edward Island, Charlottetown, PEI C1A 4P3, Canada; Email: mmcculloch@upei.ca (M.W.B.M.); bhaltli@upei.ca (B.H.); dmarchbank@upei.ca (D.H.M.); 2 Nautilus Biosciences Canada, Inc., Charlottetown, PEI C1A 4P3, Canada

**Keywords:** pseudopteroxazoles, semi-synthesis, *Mycobacteriumtuberculosis*, antibiotic-resistance

## Abstract

Pseudopterosins and pseudopteroxazole are intriguing marine natural products that possess notable antimicrobial activity with a commensurate lack of cytotoxicity. New semi-synthetic pseudopteroxazoles, pseudopteroquinoxalines and pseudopterosin congeners along with simple synthetic mimics of the terpene skeleton were synthesized. In order to build structure-activity relationships, a set of 29 new and previously reported compounds was assessed for *in* *vitro* antimicrobial and cytotoxic activities. A number of congeners exhibited antimicrobial activity against a range of Gram-positive bacteria including *Mycobacterium* *tuberculosis* H_37_Rv, with four displaying notable antitubercular activity against both replicating and non-replicating persistent forms of *M.* *tuberculosis*. One new semi-synthetic compound, 21-((1*H*-imidazol-5-yl)methyl)-pseudopteroxazole (**7a**), was more potent than the natural products pseudopterosin and pseudopteroxazole and exhibited equipotent activity against both replicating and non-replicating persistent forms of *M.* *tuberculosis* with a near absence of *in* *vitro* cytotoxicity. Pseudopteroxazole also exhibited activity against strains of *M.* *tuberculosis* H_37_Rv resistant to six clinically used antibiotics.

## 1. Introduction

*Mycobacterium* *tuberculosis* is the causative agent of tuberculosis (TB), a disease which remains a serious threat to the global human population, causing nearly 2 million deaths and over 9 million new infections annually [1]. While the majority of TB patients can be cured using existing antibiotic treatment regimens, several challenges still exist for the treatment of TB. A key drawback to current therapies is the lengthy duration (6–9 months) required to ensure complete eradication of the disease. The long duration and associated toxicity result in poor patient compliance which contributes to the spread of TB and selects for drug-resistant forms of the disease. The need for extended TB treatment regimens is in large part due to the resistance of non-replicating persistent (NRP) subpopulations of *M.* *tuberculosis* to antibiotic treatment. The treatment of TB is further complicated by the increasing occurrence of strains resistant to multiple drugs, which account for approximately 5% of TB cases [2]. To improve the outcome of existing TB treatments, new classes of molecules active against NRP-TB and emerging drug-resistant strains are greatly needed [3,4]. Natural products represent an obvious starting point to meet this desideratum given that they have historically provided a wealth of antibiotic lead compounds which have been successfully developed into efficacious drugs [5].

**Figure 1:**
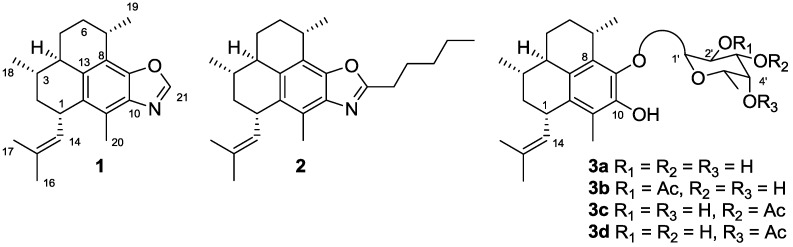
Structures of pseudopteroxazole (**1**), homopseudopteroxazole (**2**) and pseudopterosins G–J (**3a**–**d**).

The diterpenes pseudopteroxazole (**1**) and homopseudopteroxazole (**2**) (Figure 1) are trace marine natural products from *Pseudopterogorgia* *elisabethae* with reported activity against *Mycobacterium* *tuberculosis* H_37_Rv [6,7]. Despite interest in **1** by the synthetic chemistry community [8,9,10], no medicinal chemistry efforts around this scaffold were reported until our recent semi-synthesis of **1**, **2 **and 14 congeners from relatively abundant natural pseudopterosins G–J (**3a**–**3d**) [11]. In this earlier report we described activity against model mycobacteria (*M.* *smegmatis* and *M*. *diernhoferi*) and clinically relevant Gram-positive bacteria: methicillin-resistant *Staphylococcus* *aureus* (MRSA) and vancomycin-resistant *Enterococcus* *faecium* (VRE). The pseudopteroxazole pharmacophore is not known, thus an aim of the current study was to conduct a preliminary examination of structure-activity relationships (SAR), especially against *M.* *tuberculosis* H_37_Rv.

In the previous report we examined the effect of modifying the oxazole moiety in **1** by synthesizing C-21 substituted derivatives of **1** and also by preparing isopseudopteroxazoles, which are pseudopteroxazole congeners where the location of the oxazole nitrogen and oxygen atoms are inverted. We found that appending lipophilic moieties to the C-21 oxazole decreased the antimicrobial activity against model mycobacteria, MRSA and VRE, whereas isopseudopteroxazoles and their corresponding pseudopteroxazoles exhibited similar antimicrobial activity [11]. We report herein the *in* *vitro* activity of the aforementioned pseudopteroxazole compound set and new structurally related compounds against *M.* *tuberculosis* H_37_Rv and a model of NRP-TB. Given that the pseudopterosins [12,13] are also known to possess antibiotic activity against various Gram-positive bacteria [14,15,16,17] including *M.* *tuberculosis* [15], we aimed to synthesize and evaluate derivatives based on the parent aglycone scaffold of both pseudopterosins G–J and pseudopteroxazole. In this regard we have: (1) substituted the oxazole moiety in **1** with a pyrazine to generate pseudopteroquinoxaline (**5**); (2) synthesized a range of pseudopteroxazole derivatives such as 21-((1*H*-imidazol-5-yl)methyl)-pseudopteroxazole (**7a**) and the regioisomer (**7b**), which are pseudopteroxazoles with polar, amphoteric imidazole side chains that possess activity in a NRP-TB model; (3) examined the biological effect of altering the phenolic substituents on the pseudopterosins G–J aglycone (**4**); and (4) synthesized small prenylated phenol derivatives and related glycosides as mimics of the pseudopterosin/pseudopteroxazole structural core.

## 2. Results and Discussion

### 2.1. Chemistry

Pseudopteroxazoles (**1**, **2**, **22**–**33**) were synthesized as previously described [11]. The synthesis of the novel compounds is discussed below.

#### 2.1.1. Synthesis of Pseudopteroquinoxalines **5** and **6**

**Scheme 1:**
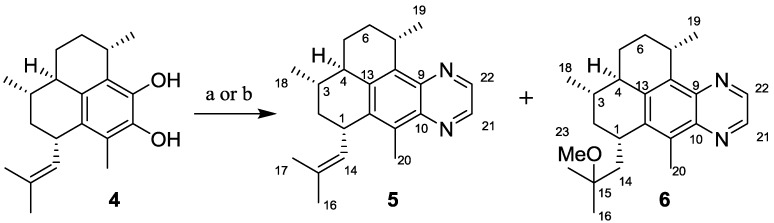
Reagents and conditions: (**a**) Ag_2_O (1.5 equiv.), NH_2_(CH_2_)_2_NH_2_, Δ, EtOH, for **5**. (**b**) (i) Dess-Martin periodinane (2 equiv.), NH_2_(CH_2_)_2_NH_2_, DCM/H_2_O/MeOH r.t.; (ii) Δ, isopropyl alcohol, for **5** and **6**.

Pseudopteroquinoxaline (**5**) was synthesized in one-pot by oxidation of the pseudopterosin G–J aglycone (**4**) [18] with Ag_2_O and condensation with ethylenediamine (Scheme 1). In an alternative synthesis, treatment of **4** with Dess-Martin periodinane in DCM/MeOH, followed by reaction with ethylenediamine yielded **5** and the tertiary ether **6** as a minor side product.

#### 2.1.2. Synthesis of Pseudopteroxazoles **7a** and **7b**

**Scheme 2:**
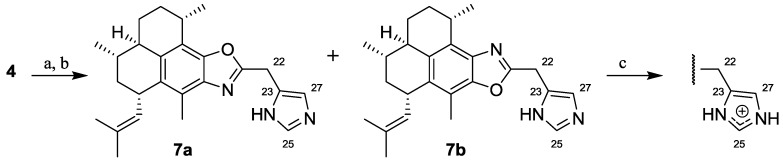
Reagents and conditions: (**a**) Ag_2_CO_3_ (1.4 equiv.), Δ, MeOH/H_2_O (10:1); (**b**) histidine (6.7 equiv. in batches), Δ; (**c**) HCO_2_H (HPLC purification).

Synthesis of the novel derivatives 21-((1*H*-imidazol-5-yl)methyl)-pseudopteroxazole (**7a**) and the regioisomer (**7b**) followed the previously reported general method utilizing the aglycone (**4**), Ag_2_O and histidine (Scheme 2). After purification by flash chromatography, a mixture of **7a** and **7b** was obtained in a 2.4:1 ratio as determined by ^1^H NMR analysis. This product regioisomer ratio differs from the ~10:1 ratio previously observed with other amino acids [11]. While the reasons behind this are under further investigation, it is conceivable that the nucleophilic imidazole attacks the *ortho*-quinone at C-10, which increases the relative rate of condensation at C-9. Separation of the regioisomers (**7a**/**7b**) proved challenging; while HPLC did not lead to peak resolution, a portion of the material was slightly enriched in **7a** (3:1 ratio) by peak shaving. This material (**7a**/**7b**) has been thoroughly and unambiguously characterized; spectra and analytical chromatograms are provided in the supporting information.

#### 2.1.3. Synthesis of Pseudopterosin Derivatives **8**–**12**

**Scheme 3:**
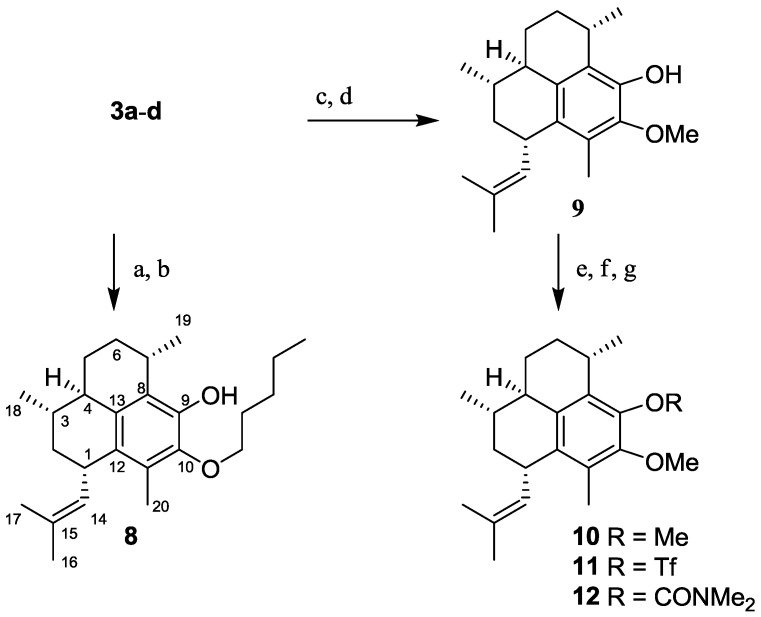
Reagents and conditions: (**a**) K_2_CO_3_, iodopentane, Δ, acetone; (**b**) HCl, Δ, MeOH; (**c**) K_2_CO_3_, MeI, Δ acetone; (**d**) HCl, Δ, MeOH; (**e**) For **10**, NaH, MeI, THF; (**f**) For **11**, Hunig’s base, Tf_2_O, DCM, 0 °C→r.t.; (**g**) For **12**, NaH, (CH_3_)_2_NCOCl, THF.

The mono-pentyl ether (**8**) derivative of the pseudopterosin G–J aglycone (**4**) was synthesized to provide a phenolic mimic of homopseudopteroxazole (**2**), given that **2** possesses a pentyl chain and was reportedly active against *M.* *tuberculosis* H_37_Rv [7]. The mono-pentyl ether (**8**) and mono-methyl ether (**9**) [19] were synthesized by alkylation of **3a**–**d** with iodopentane or iodomethane, respectively, followed by acid catalyzed hydrolysis of the fucose moiety (Scheme 3). Further substitution of the free phenol in **9** by treatment with the appropriate electrophile yielded the di-methyl ether (**10**), the triflate (**11**) and the carbamate (**12**).

#### 2.1.4. Synthesis of Pseudopterosin Mimics **14**–**20**

**Scheme 4:**
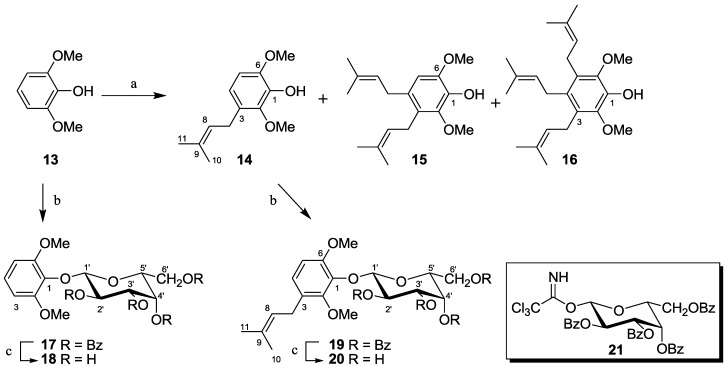
Reagents and conditions: (**a**) 2-methyl-3-buten-2-ol (1.9 equiv.), TsOH (cat.), DCM/MeOH, Δ; (**b**) **21**, BF_3_·Et_2_O,−78 °C, DCM;(**c**) K_2_CO_3_, MeOH:MTBE (5:1).

The syntheses of the prenylated aromatic mimics of pseudopterosin are shown in Scheme 4. Acid catalyzed reaction of 2,6-dimethoxyphenol (**13**) with 2-methyl-3-buten-2-ol yielded the mono-, di- and tri-prenylated derivatives (**14**, **15*** &* **16**). Compound **13** was further utilized as a model compound to develop conditions suitable for the glycosylation of these phenols. Treatment of **13** with the benzoylated glycosyl donor **21** and BF_3_·Et_2_O yielded **17**, which was deprotected with K_2_CO_3_ to give the galactoside **18** in high yield. Identical reaction sequences utilizing **14** gave the benzoylated glycoside **19** followed by the desired prenylated galactoside **20**. Attempts to glycosylate **15** were unsuccessful.

### 2.2. Antibacterial Activity

#### 2.2.1. Activity of Semi-Synthetic Pseudopteroxazoles in *M. tuberculosis* Assays

The biological activities of fifteen semi-synthetic pseudopteroxazoles and isopseudopteroxazoles are shown in Table 1. The minimum inhibitory concentrations (MICs) against *M.* *tuberculosis* H_37_Rv (ATCC 27294) were determined *in* *vitro*using the microplate Alamar blue assay (MABA) [20]. Generally substitutions at the C-21 oxazole moiety in **1** lead to congeners with reduced activity against *M.* *tuberculosis* H_37_Rv, however, three compounds (**7a**/**7b**, **22** & **25**) showed activity against *M.* *tuberculosis* H_37_Rv comparable to **1**. Semi-synthetic homopseudopteroxazole (**2**) was not active against *M.* *tuberculosis* H_37_Rv in contrast to the literature report for natural **2** isolated from *P.* *elisabethae*, which was reported to show 80% inhibition against *M.* *tuberculosis* H_37_Rv at 12.5 μg/mL (40 μM) [7]. Our result with **2** was consistent with the inactivity of other members of the series with lipophilic C-21 substituents.

**Table 1:** Antitubercular, low-oxygen-recovery assay (LORA) and cytotoxic activity of semi-synthetic pseudopteroxazoles *in* *vitro*.

Compound	MABA ^a^MIC [μg/mL] (% inh)	LORA ^b^ % inh ^c^	LORA ^b^ MIC [μg/mL]	Vero cell IC_50_ [μg/mL] (% inh)	SI ^d^
**1** (Ptx-H)	15	99.7	50	>128 (0%)	>8.6
**2** (Ptx-(CH_2_)_4_CH_3_)	>128 (6.3%)	22.6	NT	>128 (0%)	NA
**7a/7b** (Ptx-CH_2_-(1*H*-imidazol-5-yl)) ^e,f^	13	92.5	12	>128 (4%)	>9.7
**22** (*iso*-Ptx-H)	14	100.0	44	52	3.6
**23** (Ptx-(2-CH_3_O-Ph))	>82 (48%)	83.3	NT ^g^	34	NA ^h^
**24** (Ptx-(4-F-Ph))	>31 (20%)	−19.0	NT	>31 (0%)	NA
**25** (Ptx-CH_3_)	15	99.0	NT	12	0.8
**26** (Ptx-CH(CH_3_)CH_2_CH_3_)	>103 (0%)	59.1	NT	73	NA
**27** (Ptx-(CH_2_)_2_SCH_3_)	106.8	90.0	NT	>128 (0%)	>1.2
**28** (Ptx-CH_2_Ph)	>128 (28%)	13.7	NT	82	NA
**29** (Ptx-CHOHCH_3_)	53	99.8	NT	24	0.5
**30** (Ptx-(CH_2_)_2_CO_2_CH_3_)	>128 (81%)	99.1	62	31	NA
**31** (Ptx-(CH_2_)_2_CO_2_H)	95	99.1	NT	102	1.1
**32** (Ptx-(CH_2_)_2_CONH_2_)	29	99.0	NT	54	1.9
**33** (Ptx-CH_2_CONH_2_)	59	97.1	NT	45	0.8
Rifampin	0.04	NT	0.93	NT	NA
Isoniazid	0.03	NT	>128 (65%)	NT	NA
PA824	0.15	NT	NT	NT	NA
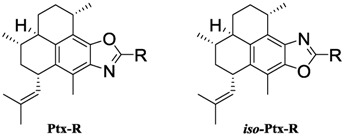					

^a^ Microplate Alamar blue assay against *M.* *tuberculosis* H_37_Rv (ATCC 27294); ^b^ Low-oxygen-recovery assay against *M.* *tuberculosis* H_37_Rv; ^c^ % inhibition at 64 μg/mL; ^d^ Selectivity Index = (Vero Cell IC_50_)/(*M.* *tuberculosis* MIC); ^e^ 3:1 ratio of regioisomers (**7a**/**7b**); ^f^**7a**/**7b** also displayed activity against MRSA (IC_50_ 3 μg/mL), VRE (IC_50_ 7.5 μg/mL), *M.* *smegmatis* (MIC 4 μg/mL) and *M.* *diernhoferi* (MIC 2 μg/mL) and was inactive against *C.* *albicans* and *P.* *aeruginosa* at 128 μg/mL; ^g^ NT = not tested; ^h^ NA = not applicable.

As a measure of potential toxicity the IC_50_ values of the pseudopteroxazoles against Vero cells [21] were determined, and the selectivity index was calculated (Vero cell IC_50_/*M.* *tuberculosis* H_37_Rv MIC). Only four semi-synthetic pseudopteroxazoles were non-toxic to Vero cells at 128 μg/mL: pseudopteroxazole (**1**), homopseudopteroxazole (**2**), the methionine derivative (**27**) and the histidine derivative (**7a**/**7b**, 4% toxicity at 128 μg/mL). Of these, two (**1** and **7a**/**7b**) exhibited antitubercular activity resulting in selectivity indices >8.6. The cytotoxicity result for **1** is comparable to that observed with “natural” pseudopteroxazole (**1**), which was reported to show no significant cytotoxicity against the NCI-60 cell line assay [6]. Interestingly, isopseudopteroxazole (**22**) displayed toxicity towards the Vero cells (IC_50_ 52 μg/mL), otherwise this regioisomer exhibited very similar antimicrobial activity to **1**.

The pseudopteroxazoles were also tested at a single point concentration (64 μg/mL) in the low-oxygen-recovery assay (LORA), a model of NRP-TB [22]. Selected active compounds were further tested to determine LORA MICs; **7a**/**7b** showed the strongest activity, with an MIC of 12 μg/mL (Table 1). As antibiotics acting on the cell wall are typically not active against NRP-TB, the LORA activity exhibited by pseudopteroxazole and several semi-synthetic congeners suggests that the target of these compounds is not the cell wall [22].

The semi-synthetic histidine derivative (**7a**/**7b**) is the most promising of the pseudopteroxazoles as it was non-toxic, exhibited potent broad-spectrum Gram-positive antibiotic activity and was the most active compound in assays against replicating and NRP-TB (MABA and LORA, respectively). While the MIC of **7a**/**7b** against *M.* *tuberculosis* H_37_Rv was moderate (13 μg/mL or 34 μM) in comparison to the first line drugs isoniazid and rifampin, its activity compares favorably to other first and second line TB treatments such as ethambutol (4.6–9.2 μM), kanamycin (2.5–10.3 μM), capreomycin (0.94–3.7 μM) and cycloserine (122–490 μM) [23]. One of the most promising attributes of **7a**/**7b** was that it showed greater potency in LORA (LORA MIC 12 μg/mL or 31 μM) compared to **1** (LORA MIC 50 μg/mL or 162 μM). Activity against NRP-TB is highly desirable given this phenotype contributes to lengthy treatment regimens leading to poor patient compliance ultimately translating into increased TB transmission rates and selection for drug-resistant strains [4]. As shortening of treatment times is a key goal of current TB chemotherapeutic research, this compound may represent a starting point for developing drugs that are more efficacious towards latent TB infections [1].

Recently a diterpene that is structurally related to pseudopterosins has been shown to possess activity against *M.* *tuberculosis* H_37_Rv and a multidrug-resistant strain [24]. These examples suggest that the semi-synthetic pseudopteroxazole congeners may also display activity against drug-resistant strains. Thus, we determined MICs of **1** against six isogenic mono-resistant *M.* *tuberculosis* H_37_Rv strains (Table 2). In our study the six strains tested were singly-resistant to a structurally diverse group of antibiotics targeting a variety of cellular targets. The aminoglycosides streptomycin and kanamycin target the 30S ribosome, the fluoroquinolone moxifloxacin targets DNA gyrase, the ansamycin antibiotic rifampin targets RNA polymerase and the small heterocyclic antibiotics isoniazid and cycloserine inhibit cell wall biosynthesis, albeit via distinct mechanisms [25,26,27]. Pseudopteroxazole exhibited virtually identical activity against wild-type and antibiotic resistant strains. While **1** was significantly less potent than rifampin and isoniazid none of the antibiotic resistant strains exhibited cross-resistance to **1**, suggesting that it may exert its antimicrobial activity via a unique mode of action.

**Table 2:** Susceptibility of mono-antibiotic resistant *M.* *tuberculosis* H_37_Rv isogenic strains to pseudopteroxazole, rifampin and isoniazid.

	MABA ^a^ MIC [μg/mL]						
Compound	H_37_Rv	RMP^r^	INH^r^	SM^r^	KM^r^	CS^r^	MOX^r^
**1**	7	8	8	14	16	14	8
Rifampin	0.03	>3.3	0.02	0.08	0.02	0.01	0.02
Isoniazid	0.03	0.12	>1.10	0.13	0.13	0.12	0.03

^a^ Microplate Alamar blue assay against wild-type *M.* *tuberculosis* H_37_Rv (H_37_Rv) and *M.* *tuberculosis* H_37_Rv isogenic strains resistant to rifampin (RMP^r^), isoniazid (INH^r^), streptomycin (SM^r^), kanamycin (KM^r^), cycloserine (CS^r^) and moxifloxacin (MOX^r^).

#### 2.2.2. Anti-Microbial Activity of Semi-Synthetic Pseudopteroquinoxalines, Pseudopterosins, and the Prenylated Mimics

The biological data of the pseudopterosin derivatives (including the pseudopteroquinoxalines) and of the prenylated aromatic mimics of pseudopterosins are summarized in Table 3. These compounds were tested *in* *vitro* against MRSA, VRE, *M.* *smegmatis* (ATCC 12051), and *M.* *diernhoferi* (ATCC 19340) using microbroth dilution antibiotic susceptibility assays. The compounds were also assessed for activity against *M.* *tuberculosis*, Vero cells and in the LORA. Natural pseudopterosins G–J (**3a**–**d**) showed the strongest activity against all pathogens, and exhibited low toxicity towards Vero cells. The mono-methyl ether (**9**) retained some activity against all pathogens; however, all other semi-synthetic derivatives showed significantly reduced activity against one or more organisms. Pseudopteroquinoxaline (**5**) was moderately active against the three mycobacteria; however, it was toxic towards the Vero cells.

**Table 3:** Antibacterial, LORA and cytotoxicity activities of pseudopterosins, pseudopteroquinoxalines and structural mimics *in* *vitro*.

	IC_50_ [μg/mL]		MIC [μg/mL] (% inh)						
Compound	MRSA ^a^	VRE ^a^	*M.* *smegmatis*^a^	*M.* *diernhoferi*^a^	*M.* *tuberculosis* ^b^ (% inh)	LORA ^c^ % inh ^d^	LORA ^c^ MIC [μg/mL]	Vero cell IC_50_ [μg/mL] (% inh)	SI ^e^
**3a–d** ^f^ (Ps G–J mixture)	<1	<1	2	2	30	97.9	NT ^g^	>128 (32%)	>4.3
**4** ^f^ (Ps G–J aglycone)	88	>128	8	8	>128 (86%)	53.1	NT ^g^	50	NA ^h^
**5** (pseudopteroquinoxaline) ^f^	>128	>128	16	64	29	99.9	NT	22	0.8
**6** (Me ether of pseudopteroquinoxaline)	NT	NT	NT	NT	84	99.9	NT	15	0.2
**8** ^f^ (Ps G–J mono-pentyl ether)	47	22	>128	>128	>128 (85%)	35.1	NT	49	NA
**9** ^f^ (Ps G–J mono-methyl ether)	9	12	4	4	30	99.7	52	>128 (26%)	>4.3
**10** ^f^ (Ps G–J di-methyl ether)	>128	25	>64	>64	>128 (63%)	77.5	NT	51	NA
**11** ^f^ (triflate)	>128	>128	>64	>64	>128 (44%)	37.5	NT	51	NA
**12** ^f^ (carbamate)	>128	70	64	>64	>128 (77%)	80.0	NT	>128 (0%)	NA
**14** ^f^ (mono-prenylated mimic)	>128	>128	128	64	>128 (24%)	−4.7	NT	44	NA
**15** ^f^ (di-prenylated mimic)	20	3	8	8	59	99.7	58	64	1.1
**16** ^f^ (tri-prenylated mimic)	>128	3	>128	8	56	79.7	NT	>128 (29%)	>2.3
**20** (galactoside of 14)	>128	>128	>128	>128	>128 (1.4%)	−24.8	NT	82	NA
Vancomycin	1.23	NT	NT	NT	NT	NT	NT	NT	NA
Rifampin	NT	0.88	4	4	0.04	NT	0.93	NT	NA
Isoniazid	NT	NT	NT	NT	0.03	NT	>128 (65%)	NT	NA
PA824	NT	NT	NT	NT	0.15	NT	NT	NT	NA

^a^ Microbroth dilution antibiotic susceptibility assay; ^b^ Microplate Alamar blue assay against *M.* *tuberculosis* H_37_Rv (ATCC 27294); ^c^ Low-oxygen-recovery assay against *M.* *tuberculosis* H_37_Rv (ATCC 27294); ^d^ % inhibition at 64 μg/mL; ^e^ Selectivity Index = (Vero Cell IC_50_)/(*M.* *tuberculosis* MIC); ^f^ No inhibitory activity was observed against *C.* *albicans* nor against *P.* *aeruginosa* at 128 μg/mL; ^g^ NT = not tested; ^h^ NA = not applicable.

The activity of the prenylated aromatic mimics is interesting: the mono-prenylated compound (**14**) was only weakly active against *M.* *smegmatis* and *M.* *diernhoferi*; the tri-prenylated compound (**16**) showed moderate activity against *M.* *diernhoferi*, and was also active against VRE and *M.* *tuberculosis*; the di-prenylated compound (**15**) was the most active as it showed good to moderate activity against all bacteria with an IC_50_ of 3 μg/mL against VRE. To provide a glycoside mimic of pseudopterosins, the galactoside derivative **20** was synthesized from **14**. This synthetic galactoside was less active than the parent prenylated mimic and no additional glycosides were synthesized following unsuccessful glycosylation attempts utilizing the di-prenylated compound (**15**). The activity of the prenylated aromatics hints at the possibility of a simpler pharmacophore than the natural diterpene skeleton. However, more work is required here as it does not necessarily follow that these mimics operate through the same mechanism of action as the pseudopterosins/pseudopteroxazoles.

#### 2.2.3. Relevance of the Use of Model *Mycobacteria*

Due to the challenges associated with cultivating *M.* *tuberculosis* (slow growth rate, biosafety risk, biosafety level 3 containment requirements) we initially used two fast growing mycobacteria, *M.* *smegmatis* and *M.* *diernhoferi*, as model organisms to evaluate the antimycobacterial activity of the compounds described herein. The usefulness of fast growing mycobacteria to detect compounds inhibitory to the growth of TB, particularly the widely used *M.* *smegmatis*, has recently been questioned. In a comprehensive study of the relative activity of 5000 compounds against *M.* *smegmatis* and *M.* *tuberculosis*, 50% of compounds active against *M.* *tuberculosis* were not detected as active against *M.* *smegmatis* [27]. Despite this disparity, the use of fast growing mycobacterial models continue to have utility as a whole cell screen against *M.* *smegmatis* identified the promising new diarylquinoline, TMC207, which is currently in Phase II–III clinical trials for the treatment of multidrug-resistant TB [1]. In the evaluation of the series of pseudopteroxazole congeners the model mycobacteria were good predictors of antimycobacterial activity (Table 1 and Table 3, and previously published data [11]). There were no instances of false-negative predictions (*i.e.*, the model organisms were insensitive to a compound which inhibited the growth of *M.* *tuberculosis*). However, there were a few instances where the model organisms predicted activity which was not mirrored by *M.* *tuberculosis* in the MABA (**4**, **27**, **28**, **31**). Interestingly, in three of these cases significant activity was observed in the LORA. These observations suggest that model mycobacteria can be a reliable predictor of *M.* *tuberculosis* activity for a particular series of molecules.

## 3. Experimental Section

### 3.1. General Experimental Procedures

The MABA, LORA and Vero cell assays were all conducted by the Institute for Tuberculosis Research following published protocols [20,21,22]. NMR spectra were obtained on a Bruker Avance III 600 MHz NMR spectrometer operating at 600 and 150 MHz for ^1^H and ^13^C, respectively. Chemical shifts (δ) are reported in ppm and were referenced to residual solvent signals: CDCl_3_ (δ_H_ 7.26; δ_C_ 77.0), (CD_3_)_2_SO (δ_H_ 2.50; δ_C_ 39.52), CD_3_OD (δ_H_ 3.31; δ_C_ 49.0), C_6_D_6_ (δ_H_ 7.15; δ_C_ 128.02). The phrase “standard work up procedure” refers to the following protocol: the organic phase is dried (MgSO_4_), filtered through glass wool, and then concentrated *in* *vacuo*. All other general experimental procedures and the syntheses of compounds **1**, **2**, **4**, & **22**–**33** are identical to those previously described [11].

### 3.2. Synthesis

#### 3.2.1. Synthesis of Pseudopteroquinoxaline (**5**)

A sample of pseudopterosin G–J (**3a**–**d**, 20 mg, 0.041 mmol) was refluxed in methanolic HCl (1.5 N, 10 mL) under N_2_ for 2.5 h. The crude mixture was then partitioned between EtOAc and H_2_O and the EtOAc phase was concentrated *in* *vacuo* to give the crude aglycone (**4**). This material was dissolved in EtOH (15 mL) and air was bubble through the sample for 10 min. Ethylenediamine (50 μL, excess) and Ag_2_O (14 mg, 0.065 mmol) were then added and the reaction was refluxed for 1.5 h. The reaction mixture was then filtered through Celite and partitioned between EtOAc and H_2_O. The EtOAc phase was subjected to the standard work up procedure and then purified by flash chromatography (Silica, hexane→MTBE gradient) to give **5** (2 mg, 0.0063 mmol, 15% over two steps). 

**5**: yellow oil; [α]^25^_D_ +103 (*c* 0.03, CHCl_3_); IR ν_max_ 2921, 2858, 1470 cm^−1^; ^1^H NMR (CDCl_3_, 600 MHz) δ 8.74 (m, 2H, H-20, H-21), 5.04 (d, 1H, *J* = 9.1 Hz, H-14), 4.11 (app. q, 1H, *J* = 8.5 Hz, H-1), 4.06 (app. q, 1H, *J* = 7.3 Hz, H-7), 2.64 (s, 3H, H-20), 2.39-2.33 (m, 1H), 2.28–2.23 (m, 1H), 2.23–2.18 (m, 1H), 2.17–2.11 (m, 1H), 1.82 (s, 3H, H-17), 1.70 (s, 3H, H-16), 1.54 (m, 1H), 1.42–1.40 (m, 2H), 1.39 (d, 3H, *J* = 6.9 Hz, H-19), 1.37–1.32 (m, 1H), 1.26–1.24 (m, 1H), 1.11 (d, 3H, *J* = 6.2 Hz, H-18) 1.14–1.09 (m, 1H); ^13^C NMR (CDCl_3_, 150 MHz) δ 142.1, 142.0, 141.9, 141.3, 141.0, 140.1, 137.0, 132.2, 129.7, 129.7, 44.3, 39.5, 37.4, 33.8, 30.7, 28.5, 26.8, 25.5, 24.7, 20.4, 17.7, 12.8; APCIMS *m/z* 321 [M + H]^+^; HRMS-ES *m*/*z* [M + H]^+^321.2313 (calcd for C_22_H_29_N_2_, 321.2325).

#### 3.2.2. Synthesis of 14,15-Dihydro-15-methoxy-pseudopteroquinoxaline (**6**)

To a solution of the pseudopterosin aglycone (**4**, 26.3 mg, 0.088 mmol) in DCM (10 mL + 100 μL H_2_O) was added Dess-Martin periodinane (68 mg, 0.16 mmol). After stirring for 15 min, MeOH (2 mL) and ethylenediamine (1 mL) were added. After another 45 min the solvent was removed *in* *vacuo*, and then isopropyl alcohol was added (20 mL). After refluxing overnight additional ethylenediamine (200 μL) was added and the solution was refluxed for a further 24 h. The reaction products were partitioned between EtOAc and H_2_O and the organic phase was subjected to the standard work up procedure to give an orange brown gum (31.8 mg). Purification by flash chromatography (diol modified silica, hexane→MTBE gradient) yielded the quinoxaline (**5**, 4.6 mg, 16%) and the methyl ether (**6**, 1.6 mg, 5%). 

**6**: amorphous semi solid; [α]^25^_D_ −5.0 (*c* 0.08, CHCl_3_); IR ν_max_ 2925, 2866, 1470, 1079 cm^−1^; ^1^H NMR (CDCl_3_, 600 MHz) δ 8.76 (s, 2H, H-20, H-21), 3.89 (app. q, 1H, *J* = 6.9 Hz), 3.71 (m, 1H), 3.20 (s, 3H, OMe), 2.79 (s, 3H, H-20), 2.49 (m, 1H), 2.21–2.15 (m, 3H), 1.89 (dd, 1H, *J* = 9.5, 14.5 Hz, H-14-a), 1.68–1.62 (m, 2H), 1.54–1.50 (m, 1H), 1.41 (d, 3H, *J* = 6.9 Hz, H-19), 1.27 (s, 6H), 1.26 (m, 2H), 1.13 (d, 3H, *J* = 6.5 Hz); ^13^C NMR (CDCl_3_, 150 MHz) δ 144.7, 142.3, 141.8, 141.6, 141.3, 140.4, 136.5, 129.8, 75.3 (C-15), 49.2 (C-23), 48.6 (C-14), 42.3, 38.2, 34.7, 32.2, 30.3, 29.4, 25.8, 25.7, 25.4, 24.0, 21.0. 12.6; APCIMS *m/z* 353 [M + H]^+^; HRMS-ES *m*/*z* [M + H]^+^353.2582 (calcd for C_23_H_33_N_2_O, 353.2587).

#### 3.2.3. Synthesis of 21-((1*H*-Imidazol-5-yl)methyl)-pseudopteroxazole (**7a**) and 21-((1*H*-Imidazol-5-yl)methyl)-isopseudopteroxazole (**7b**)

The pseudopteroxazole C-21 (1*H*-imidazol-4-yl)methyl derivatives (**7a** and **7b**) were synthesized from the pseudopterosin aglycone (**4**, 193 mg, 0.64 mmol), Ag_2_CO_3_ (1.4 equiv.) and histidine (6.7 equiv.) following the previously reported general procedure [11]. After purification by flash chromatography (diol modified silica, hexane→MTBE gradient) the product was obtained in 23% isolated yield (57.5 mg, 2.4:1 ratio of **7a**/**7b**). A portion of this material was subjected to RP-HPLC (Phenomenex, phenylhexyl, 5 μm, 250 × 10 mm, 2.9 mL/min) eluted with MeOH:H_2_O:HCO_2_H (70:30:0.1). While the regioisomers eluted as one asymmetric peak (19.8 to 20.7 min), peak shaving lead to the isolation of an enriched fraction (3:1 ratio of normal to inverse regioisomer), which was the material used for biological evaluation. 

**7a**/**7b** (3:1 ratio): orange immobile oil; [α]^25^_D_ +129.4 (*c* 0.09, CHCl_3_); IR ν_max_ 2948, 2921, 2856, 1446, 1085 cm^−1^; ^1^H and ^13^C NMR see Supplementary Table S1. APCIMS *m/z* 390 [M + H]^+^; MSMS spectrum see Supplementary Figure S9; HRMS-ES *m*/*z* [M + H]^+^ 390.2549 (calcd for C_25_H_32_N_3_O, 390.2540).

#### 3.2.4. Synthesis of 10-Pentoxy-pseudopterosin G–J Aglycone (**8**)

A solution of pseudopterosins G–J (**3a**–**d**, 102 mg, 0.2 mmol), K_2_CO_3_ (1 g, excess) and iodopentane (2 mL, excess) in anhydrous acetone was refluxed under N_2_ for 18 h. The products were then partitioned between EtOAc and H_2_O and subjected to the standard work up procedure to give an orange/brown oil (127 mg). This crude product was then dissolved in 1.5 M HCl in MeOH (10 mL) and refluxed under N_2_ for 2 h. The products were partitioned between EtOAc and H_2_O and the organic subjected to the standard work up procedure to yield the crude product, which was then purified by flash chromatography (C18, H_2_O→MeOH gradient) to yield the pentyl ether (**8**) (40.3 mg, 0.11 mmol, 52% over 2 steps). 

**8**: immobile oil; [α]^25^_D_ +71.6 (*c* 0.787, CHCl_3_); IR ν_max_ 3523, 2924, 2857, 1456, 1056 cm^−1^; ^1^H NMR (CDCl_3_, 600 MHz) δ 5.70 (s, 1H), 4.97 (d, 1H, *J* = 9.2 Hz), 3.84 (m, 1H), 3.75 (m, 1H), 3.67 (m, 1H), 3.17 (m, 1H), 2.17 (m, 1H), 2.07 (s, 3H), 2.06–2.02 (m, 2H), 1.97 (m, 1H), 1.80 (m, 2H), 1.73 (s, 3H), 1.68 (s, 3H), 1.46 (m, 2H), 1.40 (m, 2H), 1.34–1.31 (m, 1H), 1.30 (d, 3H, *J* = 6.8 Hz), 1.23–1.21 (m, 2H), 1.03 (d, 3H, *J* = 6.0 Hz), 0.95 (m, 1H), 0.95 (t, 3H, 7.2 Hz); ^13^C NMR (CDCl_3_, 150 MHz) δ 144.8, 142.8, 135.6, 131.3, 129.8, 128.3, 126.7, 125.5, 73.6, 44.7, 40.2, 37.0, 34.0, 32.1, 30.1, 28.8, 28.2, 27.8, 25.4, 23.0, 22.6, 20.0, 17.5, 14.0, 12.7; APCIMS *m/z* 371 [M + H]^+^; HRMS-ES *m*/*z* [M + H]^+^ 371.2942 (calcd for C_25_H_39_O_2_, 371.2945).

#### 3.2.5. Synthesis of 9,10-Dimethoxy-pseudopterosin G–J Aglycone (**10**)

A stirred solution of **9** [19] (25.5 mg, 0.083 mmol) in dry THF (5 mL), under N_2_, was allowed to react with an excess of NaH for 2 h. Iodomethane (200 μL, excess) was then added and the solution was left stirring at room temperature overnight. The reaction was then carefully quenched with MeOH (1 mL), and excess iodomethane was removed under a stream of N_2_. The products were then partitioned between EtOAc and H_2_O. The EtOAc fraction was subjected to the standard work up procedure to give the crude product which was purified by flash chromatography (silica, hexane→MTBE gradient) to yield the desired dimethyl ether (**10**) (18.2 mg, 0.055 mmol, 67%). 

**10**: amorphous solid; [α]^25^_D_ +69.5 (*c* 0.573, CHCl_3_); IR ν_max_ 2924, 2861, 1460, 1069 cm^−1^; ^1^H NMR (CDCl_3_, 600 MHz) δ 4.99 (d, 1H, *J* = 9.2 Hz), 3.88 (s, 3H), 3.81 (s, 3H), 3.73 (q, 1H, *J* = 8.8 Hz), 3.26 (m, 1H), 2.15–2.05 (m, 3H), 2.11 (s, 3H), 2.00 (m, 1H), 1.76 (br s, 3H), 1.71 (br s, 3H), 1.39 (m, 1H), 1.31–1.23 (m, 2H), 1.28 (d, 3H, *J* = 6.9 Hz), 1.07 (d, 3H, *J* = 6.0 Hz), 0.98 (m, 1H); ^13^C NMR (CDCl_3_, 150 MHz) δ 149.4, 149.0, 135.3, 134.0, 133.0, 130.9, 128.5, 128.4, 60.1, 59.8, 44.0, 40.1, 37.3, 34.0, 31.3, 28.3, 27.5, 25.4, 24.3, 20.0, 17.5, 12.1; APCIMS *m/z* 329 [M + H]^+^; HRMS-ES *m*/*z* [M + H]^+^ 329.2459 (calcd for C_22_H_33_O_2_, 329.2475).

#### 3.2.6. Synthesis of 9-Trifluoromethylsulfonyloxy-10-methoxy-pseudopterosin G–J Aglycone (**11**)

Triflic anhydride (6 mmol) in DCM was added to a stirred, ice-cooled solution of **9** (178 mg, 0.56 mmol) and Hunig’s base (2 mL) in dry toluene (8 mL). The reaction was stirred under N_2_, and allowed to warm to room overnight before being partitioned between DCM and aqueous HCl (1 N). The organic phase was subjected to the standard work up procedure to yield the crude triflate; purification was achieved by flash chromatography (silica, hexane→EtOAc gradient) to yield the triflate (**11**, 177 mg, 0.4 mmol, 70%, ~90% pure by ELSD LCMS). A portion of the product was further purified by RP-HPLC (Phenomenex, phenylhexyl, 5 μm, 250 × 10 mm, 4.0 mL/min) using a gradient of MeOH/H_2_O (9:1 for 1 min, then to 10:0 over 1–4 min; eluted across 9.9 to 10.25 min). 

**11**: amorphous solid; [α]^25^_D_ +62.8 (*c* 0.205, CHCl_3_); IR ν_max_ 2928, 2869, 1416, 1206, 1139 cm^−1^; ^1^H NMR (CDCl_3_, 600 MHz) δ 4.93 (d, 1H, *J* = 9.3 Hz), 3.74 (s, 3H), 3.72 (m, 1H,), 3.23 (dd, 1H, *J* = 7.2, 14.6 Hz), 2.18–2.06 (m, 3H), 2.12 (s, 3H), 2.01 (m, 1H), 1.74 (br s, 3H), 1.70 (br s, 3H), 1.37 (m, 1H), 1.26–1.21 (m, 2H), 1.25 (d, 3H, *J* = 6.9 Hz), 1.04 (d, 3H, *J* = 5.9 Hz), 0.97 (m, 1H); ^13^C NMR (CDCl_3_, 150 MHz) δ 147.9, 140.1, 139.7, 136.7, 132.6, 129.7 (2 × C), 129.5, 118.7 (q, *J* = 320 Hz), 117.6, 60.8, 44.0, 39.8, 37.4, 33.9, 30.9, 28.6, 27.0, 25.4, 23.3, 19.8, 17.6, 12.5; APCIMS *m/z* 447 [M + H]^+^; HRMS-ES *m*/*z* [M + H]^+^ 447.1804 (calcd for C_22_H_30_F_3_O_4_S, 447.1811).

#### 3.2.7. Synthesis of 9-Dimethylcarbamoyloxy-10-methoxy-pseudopterosin G–J Aglycone (**12**)

A stirred solution of **9** (25.5 mg, 0.083 mmol) in dry THF (5 mL), under N_2_, was allowed to react with an excess of NaH for 2 h. Dimethylcarbamoyl chloride (200 μL, excess) was then added and the solution was stirred overnight. The reaction was then carefully quenched with MeOH (1 mL) and the products were portioned between EtOAc and H_2_O. The EtOAc fraction was subjected to the standard work up procedure to give the crude product which was purified by flash chromatography (silica, hexane→MTBE gradient) to yield the carbamate (**12**, 13.7 mg, 0.035 mmol, 43%). 

Carbamate (**12**): amorphous solid; [α]^25^_D_ +110.2 (*c* 0.07, CHCl_3_); IR ν_max_ 2924, 2861, 1723 (CO), 1451, 1386, 1164 cm^−1^; ^1^H NMR (CDCl_3_, 600 MHz) δ 4.97 (d, 1H, *J* = 9.2 Hz), 3.70 (s, 3H), 3.71–3.67 (m, 1H), 3.16 (s, 3H), 3.04 (s, 3H), 3.03 (m, 1H), 2.14–2.10 (m, 2H), 2.07 (s, 3H), 2.07–2.02 (m, 2H), 1.98–1.94 (m, 1H) 1.73 (br s, 3H), 1.68 (br s, 3H), 1.34–1.16 (m, 6H), 1.01 (d, 3H, *J* = 6.1 Hz), 0.99 (m, 1H); ^13^C NMR (CDCl_3_, 150 MHz) δ 154.6, 148.6, 140.9, 136.4, 135.3, 130.6, 129.6, 128.7, 128.3, 60.5, 44.2, 40.0, 37.3, 36.8, 36.4, 33.7, 31.5, 28.6, 27.3, 25.4, 23.8, 19.9, 17.5, 12.3; APCIMS *m/z* 386 [M + H]^+^; HRMS-ES *m*/*z* [M + Na]^+^ 408.2505 (calcd for C_24_H_35_NO_3_Na, 408.2509).

#### 3.2.8. Prenylation of 2,6-Dimethoxyphenol: Synthesis of **14**, **15** *&* **16**

A solution of 2-methyl-3-buten-2-ol (640 mg, 7.4 mmol) in DCM (3 mL) was added dropwise to a stirred mixture of 2,6-dimethoxyphenol (**13**, 612 mg, 3.97 mmol) and TsOH (19 mg, cat) in DCM/MeOH (3:1, 30 mL). After stirring for 96 h at room temperature the solution was refluxed for 20 h and then partitioned between H_2_O and EtOAc. The organic phase was subjected to the standard work up procedure to yield a crude oil (793 mg) which was subjected to flash chromatography (C18, H_2_O→MeOH gradient) to yield the mono-prenylated product (**14**, 239 mg, 1.08 mmol, 27%), the di-prenylated product (**15**, 96 mg, 0.33 mmol, 8%), and the triprenylated product (**16**, 20 mg, 0.055 mmol, 1.4%) along with recovered starting material (321 mg, 52%).

2,6-Dimethoxy-3-(3-methylbut-2-enyl)phenol (**14**): oil; IR ν_max_ 3456, 2931, 2835, 1493, 1288, 1090 cm^−1^; ^1^H NMR (CDCl_3_, 600 MHz) δ 6.63 (d, 1H, *J* = 8.5 Hz), 6.60 (d, 1H, *J* = 8.4 Hz), 5.53 (s, 1H, OH), 5.27 (m, 1H, H-8), 3.863 (s, 3H, OMe), 3.861 (s, 3H, OMe), 3.30 (d, 2H, *J* = 7.3 Hz); 1.73 (s, 6H, H-10 & H-11); ^13^C NMR (CDCl_3_, 150 MHz) δ 145.9, 145.2, 138.5, 132.0, 127.8, 123.1, 119.1, 106.4, 60.4, 56.2, 28.0, 25.7, 17.7; APCIMS *m/z* 223 [M + H]^+^; HRMS-ES *m*/*z* [M + Na]^+^ 245.1142 (calcd for C_13_H_18_O_3_Na, 245.1148).

2,6-Dimethoxy-3,4-bis(3-methylbut-2-enyl)phenol (**15**): pale yellow oil; IR ν_max_ 3440, 2964, 2912, 1854, 1497, 1309, 1116 cm^−1^; ^1^H NMR (CDCl_3_, 600 MHz) δ 6.50 (s, 1H, H-5), 5.23 (m, 1H, olefinic), 5.07 (m, 1H, olefinic), 3.85 (s, 3H, OMe), 3.83 (s, 3H, OMe), 3.32 (d, 2H, *J* = 6.5 Hz), 3.25 (d, 2H, *J* = 6.5 Hz), 1.77 (s, 3H), 1.75 (s, 3H), 1.71 (s, 3H), 1.68 (s, 3H). ^13^C NMR (CDCl_3_, 150 MHz) δ 145.5, 145.4, 136.7, 132.1, 131.0 (2 × C), 126.0, 123.7, 123.4, 107.6, 60.6, 56.1, 31.4, 25.7, 25.6, 25.1, 17.9, 17.8; APCIMS *m/z* 291 [M + H]^+^; HRMS-ES *m*/*z* [M + Na]^+^ 313.1763 (calcd for C_18_H_26_O_3_Na, 313.1774).

2,6-Dimethoxy-3,4,5-tris(3-methylbut-2-enyl)phenol (**16**): colorless oil; IR ν_max_ 3400, 2964, 2912, 2855, 1456, 1087 cm^−1^; ^1^H NMR (CDCl_3_, 600 MHz) δ 5.49 (s, 1H, OH), 5.09 (m, 2H, olefinic), 4.97 (m, 1H, olefinic), 3.80 (s, 6H, OMe), 3.32 (d, 4H, *J* = 6.5 Hz), 3.23 (d, 2H, *J* = 6.0 Hz), 1.74 (s, 6H), 1.68 (broad overlapping singlets, 12H). ^13^C NMR (CDCl_3_, 150 MHz) δ 144.2, 140.1, 131.2, 131.0, 130.7, 129.5, 123.9, 123.8, 60.9, 27.8 (2 × C), 25.6, 25.55, 25.53, 17.90, 17.91; APCIMS *m/z* 359 [M + H]^+^; HRMS-ES *m*/*z* [M + Na]^+^ 381.2409 (calcd for C_23_H_34_O_3_Na, 381.2400).

#### 3.2.9. Synthesis of the Glycosyl Donor 2,3,4,6-Tetra-*O*-benzoyl-β-D-galactopyranosyl Trichloroacetimidate (**21**)

The glycosyl donor was synthesized from 2,3,4,6-tetra-*O*-benzoyl-α-D-galactopyranosyl bromide, which was freshly prepared using a previously described method [28]. The glycosyl bromide (2.05 g, 3.11 mmol) was hydrolyzed and subsequently reacted with trichloroacetonitrile according to existing methodology to provide the glycosyl donor **21** (704 mg, 0.951 mmol, 31% over two steps) [29].

#### 3.2.10. Synthesis of 2,6-Dimethoxyphenol-2,3,4,6-tetra-**O**-benzoyl-β-D-galactopyranoside (**17**)

2,6-Dimethoxyphenol (**13**, 30.0 mg, 0.195 mmol) and freshly prepared benzoylated glycosyl donor (**21**, 151.0 mg, 0.229 mmol) were dissolved in 8 mL anhydrous DCM and stirred with 3Å molecular sieves (500 mg) under a N_2_ atmosphere for 20 min. Afterwards, the mixture was cooled to −78 °C and BF_3_·Et_2_O (0.206 mmol) was added. After stirring for 2.5 h at −78 °C, the glycosyl donor was completely consumed as indicated by TLC. The reaction was quenched with Et_3_N (100 μL, excess), filtered, diluted with EtOAc, and partitioned with H_2_O. The EtOAc phase was recovered and concentrated *in* *vacuo* to provide the crude galactoside. Purification by flash chromatography (silica, hexane→MTBE gradient), yielded **17** (101.9 mg, 0.139 mmoles, 71%). 

**17**: white solid; [α]^25^_D_ +67.4 (*c* 0.1917, CH_2_Cl_2_); IR ν_max_ 3065, 2962, 2939, 2838, 1726, 1601, 1479, 1258, 1109, 1069, 709 cm^−1^; ^1^H NMR (C_6_D_6_, 600 MHz) δ 8.11 (d, 2H, *J* = 7.8 Hz), 8.10 (d, 2H, *J* = 7.8 Hz), 8.07 (d, 2H, *J* = 7.8 Hz), 8.00 (d, 2H, *J* = 7.8 Hz), 7.10 (t, 1H, *J* = 7.5 Hz), 7.06 (t, 1H, *J* = 7.5 Hz), 7.03 (t, 2H, *J* = 7.7 Hz), 7.01 (t, 1H, *J* = 7.5 Hz), 6.92 (t, 2H, *J* = 7.6 Hz), 6.91 (t, 2H, *J* = 7.6 Hz), 6.85 (t, 1H, *J* = 7.5 Hz), 6.78 (t, 1H, *J* = 8.4 Hz, H-4), 6.73 (t, 2H, *J* = 7.7 Hz), 6.60 (dd, 1H, *J* = 10.4, 7.9 Hz, H-2′), 6.23 (d, 2H, *J* = 8.4 Hz, H-3), 6.14 (dd, 1H, *J* = 3.6, 1.2 Hz, H-4′), 5.80 (dd, 1H, *J* = 10.4, 3.6 Hz, H-3′), 5.44 (d, 1H, *J* = 7.9 Hz, H-1′), 4.67 (dd, 1H, *J* = 11.3, 6.6 Hz, H-6′), 4.36 (dd, 1H, *J* = 11.3, 6.6 Hz, H-6′), 3.66 (app. ddd, 1H, *J* = 6.6, 1.2 Hz, H-5′), 3.25 (s, 6H, OMe); ^13^C NMR (C_6_D_6_, 150 MHz) δ 165.9, 165.8, 165.7, 165.4, 153.9 (C-2), 136.1 (C-1), 133.2, 133.1, 133.0, 132.7, 130.8, 130.5, 130.3, 130.1, 130.1, 130.0, 129.7, 129.6, 128.8, 128.6, 128.4, 128.3, 124.6 (C-4), 106.1 (C-3), 103.1 (C-1′), 72.7 (C-3′), 71.9 (C-5′), 71.4 (C-2′), 68.8 (C-4′), 62.3 (C-6′), 55.9 (OMe); HRMS-ES *m/z* [M + Na]^+^ 755.2088 (calcd for C_42_H_36_O_12_Na, 755.2099).

#### 3.2.11. Synthesis of 2,6-Dimethoxyphenol-β-D-galactopyranoside (**18**)

Compound **17** (40.3 mg, 55.0 μmol) was stirred with K_2_CO_3_ (38.8 mg) in 3 mL MeOH:MTBE (5:1) for 20 h. After deprotection was completed, as indicated by TLC (silica, hexane/MTBE), the reaction mixture was diluted with H_2_O and desalted by solid phase extraction (C18, 1:19 MeOH:H_2_O). The product was eluted with MeOH and purified by flash chromatography (C18, H_2_O→MeOH gradient) to provide **18** (12.5 mg, 39.5 μmol, 72%). 

**18**: colorless solid; [α]^25^_D_ −15.9 (*c* 0.8333, CH_2_Cl_2_); IR ν_max_ 3388, 3008, 2940, 2841, 1599, 1480, 1256, 1108, 1072 cm^−1^; ^1^H NMR ((CD_3_)_2_SO, 600 MHz) δ 6.97 (t, 1H, *J* = 8.4 Hz, H-4), 6.66 (d, 2H, *J* = 8.4 Hz, H-3), 4.83 (d, 1H, *J* = 7.6 Hz, H-1′), 4.79–4.74 (m, 2H, C-2′/3′-OH), 4.50-4.44 (m, 2H, C-4′/6′-OH), 3.74 (s, 6H, OMe), 3.67 (app. t, 1H, *J* = 3.3 Hz, H-4′), 3.56–3.52 (m, 1H, H-2′), 3.56–3.52 (m, 1H, H-6′), 3.37–3.33 (m, 1H, H-3′), 3.36–3.32 (m, 1H, H-6′), 3.27 (app. t, 1H, *J* = 6.3 Hz, H-5′); ^13^C NMR ((CD_3_)_2_SO, 150 MHz) δ 153.0 (C-2), 134.9 (C-1), 123.7 (C-4), 106.6 (C-3), 103.5 (C-1′), 75.5 (C-5′), 73.2 (C-3′), 71.4 (C-2′), 67.9 (C-4′), 60.1 (C-6′), 56.4 (OMe); HRMS-ES *m/z* [M + Na]^+^ 339.1043 (calcd for C_14_H_20_O_8_Na, 339.1050).

#### 3.2.12. Synthesis of 2,6-Dimethoxy-3-(3-methylbut-2-enyl)phenol-2,3,4,6-tetra-*O*-benzoyl-β-D-galactopyranoside (**19**)

The glycosylation procedure, described for the synthesis of **17**, was repeated using the benzoylated glycosyl donor (**21**, 175.6 mg, 0.266 mmol) and **14** (44.6 mg, 0.201 mmol) as the glycosyl accepting substrate. The reaction crude was separated by flash chromatography (silica, hexane→MTBE gradient) to provide **19** (149.1 mg, 0.186 mmol, 93%). 

**19**: pale yellow solid; [α]^25^_D_ +75.9 (*c* 0.225, CH_2_Cl_2_); IR ν_max_ 3063, 2966, 2936, 1726, 1602, 1493, 1451, 1094, 1069, 709 cm^−1^; ^1^H NMR (CD_3_OD, 600 MHz) δ 8.13 (d, 2H, *J* *=* 7.7 Hz), 7.90 (d, 4H, *J* *=* 7.8 Hz), 7.75 (d, 2H, *J* *=* 7.8 Hz), 7.67 (t, 1H, *J* *=* 7.7 Hz), 7.57 (t, 1H, *J* *=* 7.7 Hz), 7.54 (t, 2H, *J* *=* 7.7 Hz), 7.51 (t, 1H, *J* *=* 7.7 Hz), 7.45 (t, 1H, *J* *=* 7.6 Hz), 7.40 (t, 2H, *J* *=* 7.7 Hz), 7.36 (t, 2H, *J* *=* 7.7 Hz), 7.25 (t, 2H, *J* *=* 7.7 Hz), 6.80 (d, 1H, *J* = 8.6 Hz, H-4), 6.54 (d, 1H, *J* = 8.6 Hz, H-5), 6.03 (dd, 1H, *J* = 10.3, 8.0 Hz, H-2′), 6.00 (app. d, 1H, *J* = 3.4 Hz, H-4′), 5.78 (dd, 1H, *J* = 10.3, 3.4 Hz, H-3′), 5.64 (d, 1H, *J* = 8.0 Hz, H-1′), 5.14 (app. t, 1H, *J* = 7.4 Hz, H-8), 4.58 (dd, 1H, *J* = 10.8, 7.0 Hz, H-6′), 4.54 (app. dd, 1H, *J* = 7.0, 4.9 Hz, H-5′), 4.47 (dd, 1H, *J* = 10.8, 4.9 Hz, H-6′), 3.75 (s, 3H, C2-OMe), 3.53 (s, 3H, C6-OMe), 3.17-3.09 (m, 2H, H-7), 1.67 (s, 3H, H-10), 1.64 (s, 3H, H-11); ^13^C NMR (CD_3_OD, 150 MHz) δ 167.5, 167.3, 167.1, 166.8, 152.7 (C-2), 152.6 (C-6), 139.9 (C-1), 134.9, 134.6, 134.5, 134.4, 134.3, 132.9, 131.0, 131.0, 130.9, 130.7, 130.7, 130.6, 130.6, 130.5, 130.2 (C-9), 129.9, 129.6, 129.6, 129.5, 129.5, 129.1 (C-3), 129.0, 128.8, 128.7, 125.9 (C-4), 124.4 (C-8), 109.0 (C-5), 103.2 (C-1′), 73.6 (C-3′), 73.0 (C-5′), 72.3 (C-2′), 70.2 (C-4′), 63.7 (C-6′), 61.7 (2-OMe), 56.6 (6-OMe), 29.0 (C-7), 25.9 (C-10), 17.9 (C-11); HRMS-ES *m/z* [M + Na]^+^ 823.2754 (calcd for C_47_H_44_O_12_Na, 823.2725).

#### 3.2.13. Synthesis of 2,6-Dimethoxy-3-(3-methylbut-2-enyl)phenol-β-D-galactopyranoside (**20**)

The deprotection procedure, described for the synthesis of **18**, was repeated with **19** (38.5 mg, 48.1 μmol) and K_2_CO_3_ (33.4 mg). The deprotected glycoside was purified with the method described for **17** to yield 20 (13.9 mg, 36.2 μmol, 75%). 

**20**: colorless solid; [α]^25^_D_ −6.44 (*c* 0.6917, CH_3_OH); IR ν_max_ 3392, 2967, 2914, 1602, 1494, 1463, 1442, 1090 cm^−1^; ^1^H NMR ((CD_3_)_2_SO, 600 MHz) δ 6.79 (d, 1H, *J* = 8.5 Hz, H-4), 6.70 (d, 1H, *J* = 8.5 Hz, H-5), 5.20 (app. t, 1H, *J* = 7.3 Hz, H-8), 4.96 (d, 1H, *J* = 4.5 Hz, C-2′-OH), 4.92 (d, 1H, *J* = 7.6 Hz, H-1′), 4.80 (d, 1H, *J* = 5.3 Hz, C-3′-OH), 4.49 (m, 1H, C-4′-OH), 4.47 (m, 1H, C-6′-OH), 3.76 (s, 3H, C6-OMe), 3.72 (s, 3H, C2-OMe), 3.68 (app. dd, 1H, *J* = 3.5 Hz, H-4′), 3.59–3.55 (m, 1H, H-2′), 3.55–3.52 (m, 1H, H-6′), 3.39–3.36 (m, 1H, H-3′), 3.35–3.32 (m, 2H, H-6′), 3.29 (app. t, 1H, *J* = 6.2 Hz, H-5′), 3.24–3.15 (m, 2H, H-7), 1.68 (s, 3H, H-10), 1.67 (s, 3H, H-11); ^13^C NMR ((CD_3_)_2_SO, 150 MHz) δ 151.6 (C-2), 151.1 (C-6), 138.8 (C-1), 131.3 (C-9), 127.4 (C-3), 123.6 (C-4), 123.4 (C-8), 108.9 (C-5), 103.2 (C-1′), 75.5 (C-5′), 73.2 (C-3′), 71.4 (C-2′), 67.9 (C-4′), 60.7 (6-OMe), 60.1 (C-6′), 56.5 (2-OMe), 27.7 (C-7), 25.5 (C-10), 17.6 (C-11); HRMS-ES *m/z* [M + Na]^+^ 407.1687 (calcd for C_19_H_28_O_8_Na, 407.1676).

## 4. Conclusions

In conclusion, we have synthesized a series of pseudopterosin and pseudopteroxazole derivatives, including simple prenylated aromatic diterpene mimics, and evaluated their antimicrobial activity against *M.* *tuberculosis* H_37_Rv, *M.* *smegmatis*, *M*. *diernhoferi*, MRSA and VRE. The major SAR emanating from this study pertains to the C-9 and C-10 substituents off the natural pseudopterosin-like scaffold. Variability in this region is tolerated for activity against *M.* *tuberculosis* H_37_Rv and other Gram-positive pathogens, such as MRSA and VRE. For instance, the phenolic (e.g., **9**), benzoxazole (e.g., **7a**), and quinoxaline (e.g., **5**) derivatives all retained activity, though the latter was inactive against MRSA and VRE. Appropriate substitution at the C-9/C-10 position can lead to improved activity. In terms of Vero cell activity, while pseudopterosins (**3a**–**d**) did not display toxicity, *a* *priori* pseudopteroxazoles or other non-phenolic derivatives may represent better candidates for development since pseudopterosins may potentially be metabolized to the reactive *ortho*-quinone (**4**) or related derivatives.

It is likely that pseudopterosins and pseudopteroxazoles operate through a similar, and potentially novel, mechanism of action. We have commenced studies aiming to identify the antimicrobial mechanism of action of the pseudopteroxazoles and have begun investigating the effect of modifying other regions of the scaffold on biological activity.

## Supplementary Files

Supplementary File 1: PDF-Document (PDF, 2611 KB)
