# Supplementary material for: Evaluation of Pseudopteroxazole and Pseudopterosin Derivatives against Mycobacterium tuberculosis and Other Pathogens
Source: Mar Drugs. 2012 Aug 15;10(8):1711–28. doi: 10.3390/md10081711 (PMC3447335; doi:10.3390/md10081711)

Supporting Information

Table of Contents

Figure S1. ^{1}H-NMR (600 MHz, $CDCl_3$) spectrum of **5**. **2**
Figure S2. ^{13}C-NMR (150 MHz, $CDCl_3$) spectrum of **5**. **3**
Figure S3. ^{1}H-NMR (600 MHz, $CDCl_3$) spectrum of **6**. **4**
Figure S4. ^{13}C-NMR (150 MHz, $CDCl_3$) spectrum of **6**. **5**
Figure S5. HSQC (150 MHz, $CDCl_3$) spectrum of **6**. **6**
Table S1. NMR assignments **7a/7b** mixture. **7**
Figure S6. ^{1}H-NMR (600 MHz, $CDCl_3$) spectrum of **7a/7b** mixture. **8**
Figure S7. ^{13}C-NMR (150 MHz, $CDCl_3$) spectrum of **7a/7b** mixture. **9**
Figure S8. HSQC spectrum (600 MHz, $CDCl_3$) of **7a/7b** mixture. **10**
Figure S9. MS (top) and MSMS spectra (bottom) of **7a/7b**. **11**
Figure S10. Analytical UPLC Chromatogram of **7a/7b**. (**a**) UV trace 255 nm; (**b**) ELSD trace; (**c**) Base peak mass chromatogram; (**d**) Selected ion monitoring *m/z* 390 [M + H]; (**e**) Average mass spectrum (5.15–5.45 min). **12**
Figure S11. ^{1}H-NMR (600 MHz, $CDCl_3$) spectrum of **8**. **13**
Figure S12. ^{13}C-NMR (150 MHz, $CDCl_3$) spectrum of **8**. **14**
Figure S13. ^{1}H-NMR (600 MHz, $CDCl_3$) spectrum of **10**. **15**
Figure S14. ^{13}C-NMR (150 MHz, $CDCl_3$) spectrum of **10**. **16**
Figure S15. ^{1}H-NMR (600 MHz, $CDCl_3$) spectrum of **11**. **17**
Figure S16. ^{13}C-NMR (150 MHz, $CDCl_3$) spectrum of **11**. **18**
Figure S17. ^{1}H-NMR (600 MHz, $CDCl_3$) spectrum of **12**. **19**
Figure S18. ^{13}C-NMR (150 MHz, $CDCl_3$) spectrum of **12**. **20**
Figure S19. ^{1}H-NMR (600 MHz, $CDCl_3$) spectrum of **14**. **21**
Figure S20. ^{13}C-NMR (150 MHz, $CDCl_3$) spectrum of **14**. **22**
Figure S21. ^{1}H-NMR (600 MHz, $CDCl_3$) spectrum of **15**. **23**
Figure S22. ^{13}C-NMR (150 MHz, $CDCl_3$) spectrum of **15**. **24**
Figure S23. ^{1}H-NMR (600 MHz, $CDCl_3$) spectrum of **16**. **25**
Figure S24. ^{13}C-NMR (150 MHz, $CDCl_3$) spectrum of **16**. **26**
Figure S25. ^{1}H-NMR (600 MHz, C_6D_6) spectrum of **17**. **27**
Figure S26. ^{13}C-NMR (600 MHz, C_6D_6) spectrum of **17**. **28**
Figure S27. ^{1}H-NMR (600 MHz, $(CD_3)_2SO$) spectrum of **18**. **29**
Figure S28. ^{13}C-NMR (600 MHz, $(CD_3)_2SO$) spectrum of **18**. **30**
Figure S29. ^{1}H-NMR (600 MHz, CD_3OD) spectrum of **19**. **31**
Figure S30. ^{13}C-NMR (600 MHz, CD_3OD) spectrum of **19**. **32**
Figure S31. ^{1}H-NMR (600 MHz, $(CD_3)_2SO$) spectrum of **20**. **33**
Figure S32. ^{13}C-NMR (600 MHz, $(CD_3)_2SO$) spectrum of **20**. **34**
Scheme S1. Syntheses of 2,3,4,6-tetra-*O*-benzoyl-β-d-galactopyranosyl trichloroacetimidate (**21**). **35**

Figure S1. ^{1}H-NMR (600 MHz, $CDCl_3$) spectrum of **5**.

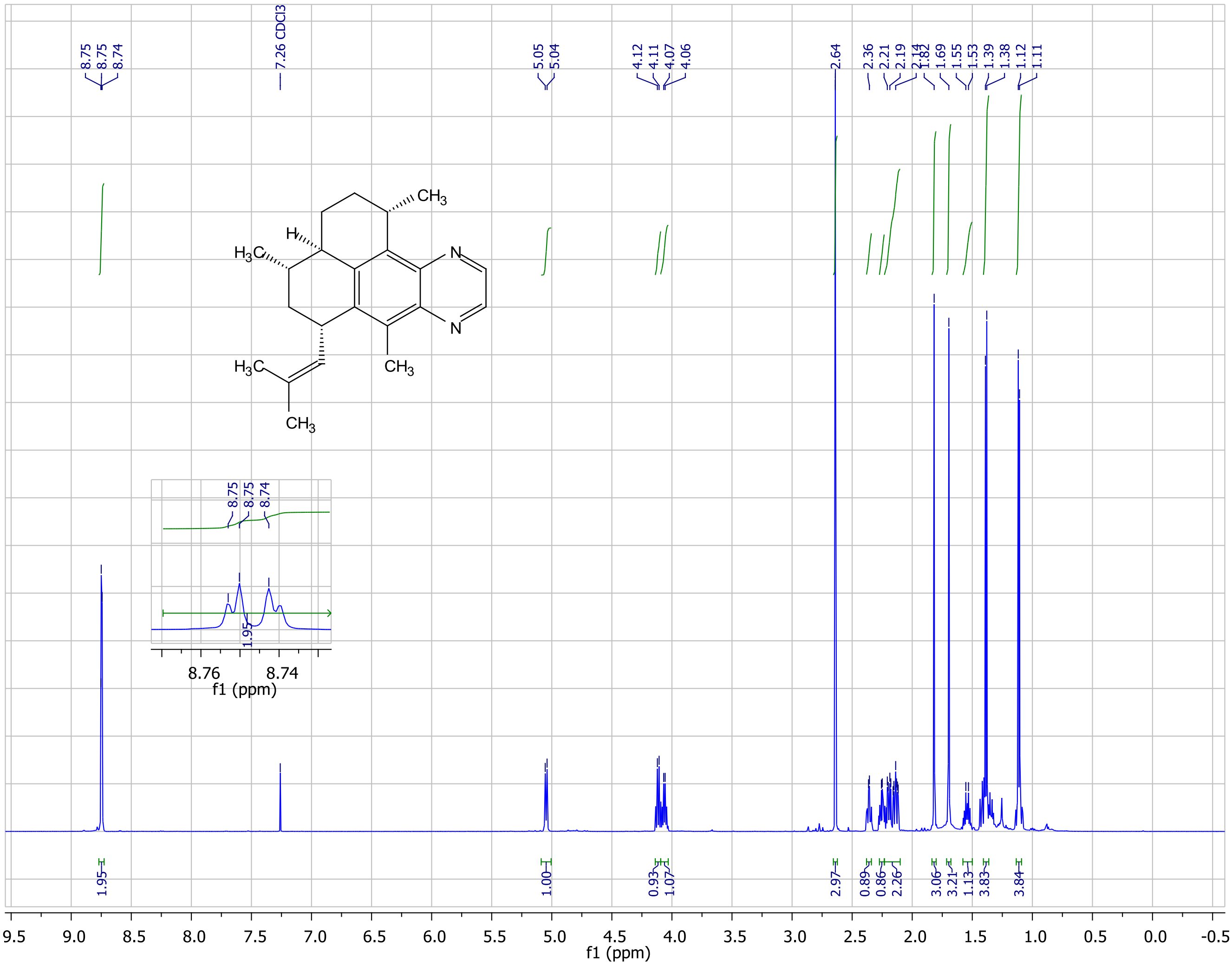

Figure S2. ^{13}C-NMR (150 MHz, $CDCl_3$) spectrum of **5**.

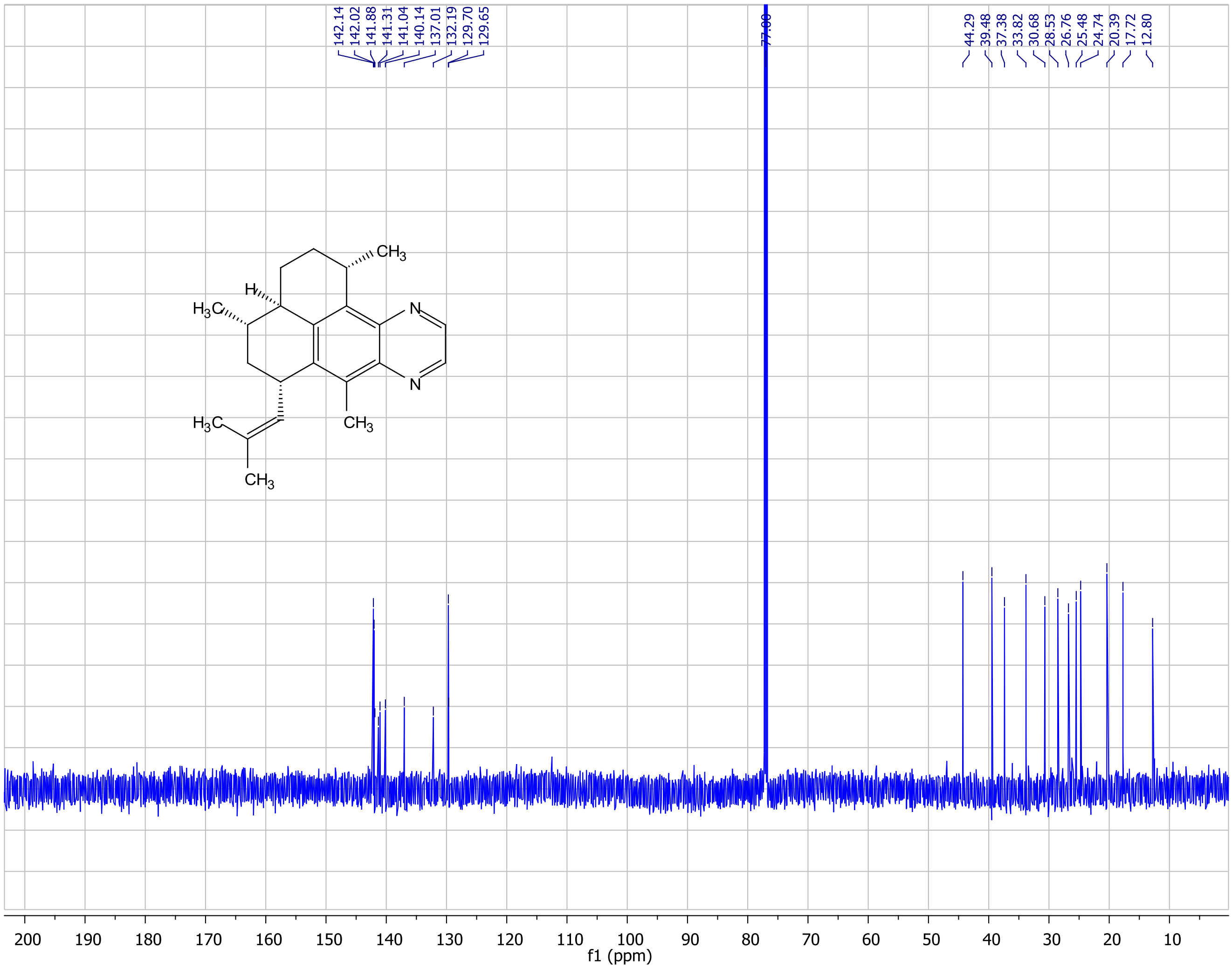

Figure S3. ¹H-NMR (600 MHz, $CDCl_3$) spectrum of **6**.

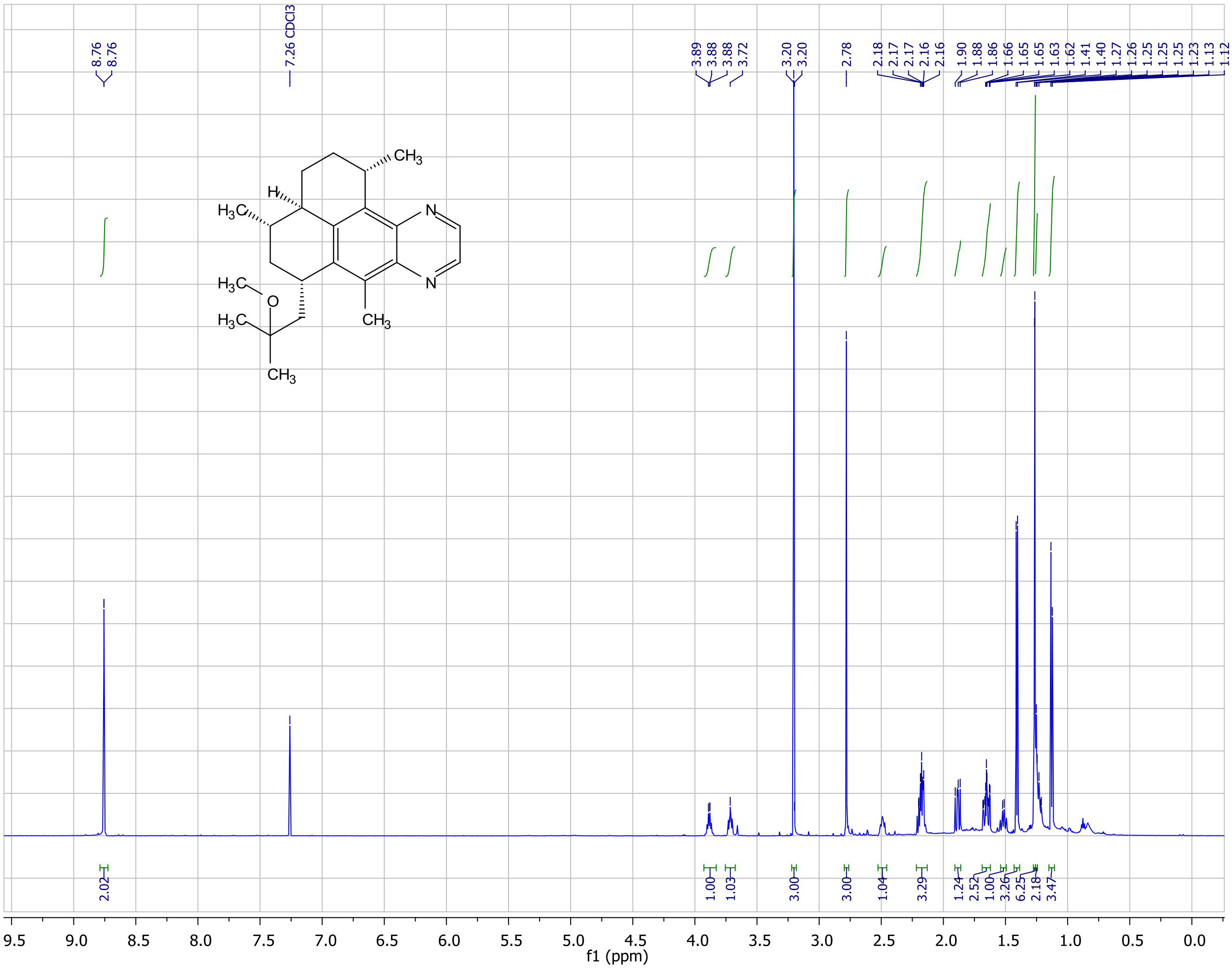

Figure S4. ^{13}C-NMR (150 MHz, $CDCl_3$) spectrum of **6**.

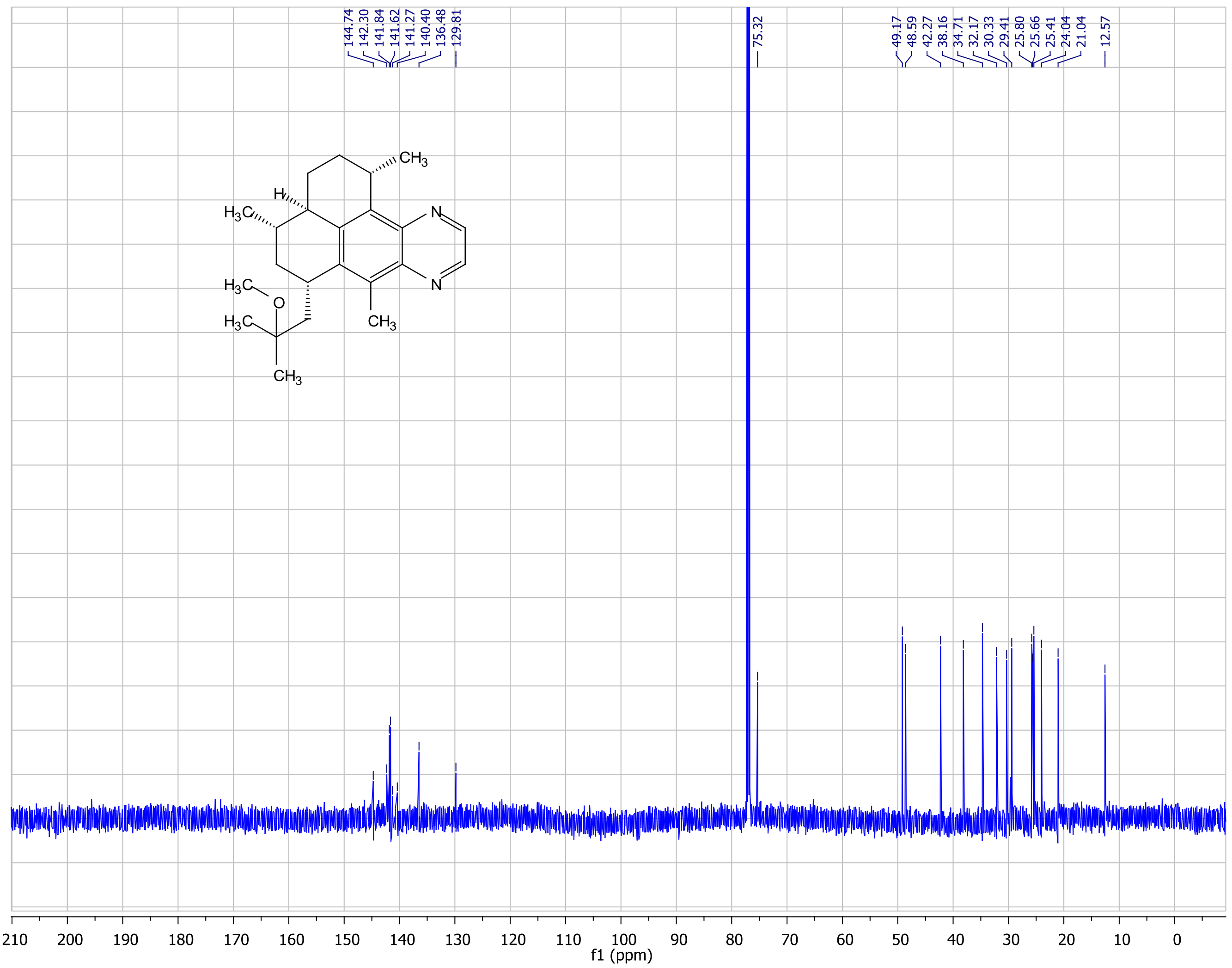

Figure S5. HSQC (150 MHz, $CDCl_3$) spectrum of **6**.

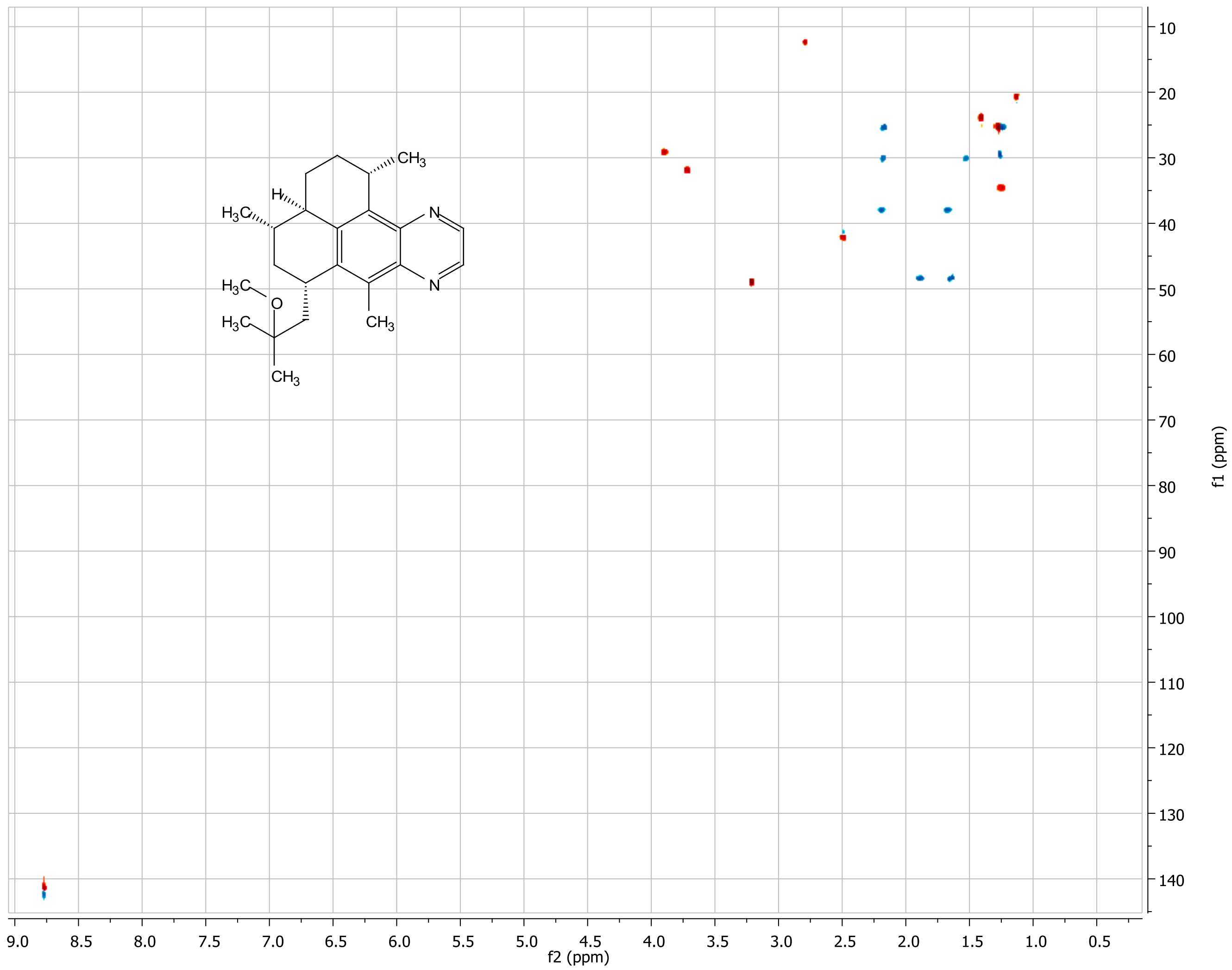

Table S1. NMR assignments **7a/7b** mixture.

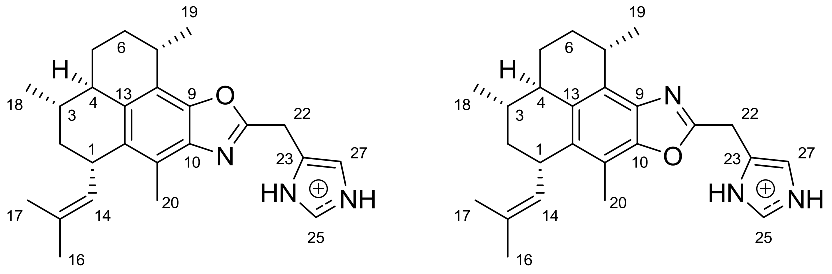

~3:1 ratio **7a**/**7b**

Position	7a [a]		7b [a]	
	δ_C	δ_H (*J* in Hz)	δ_C	δ_H (*J* in Hz)
1	36.4	3.88, m	36.7	3.88, m
2 a	40.0	2.09, m	40.0	2.09, m
b	-	1.24, m	-	1.24, m
3	34.5	1.24, m	34.4	1.24, m
4	44.67	2.20, m	44.72	2.20, m
5 a	28.0	2.13, m	28.0	2.13, m
b	-	1.04, m	-	1.04, m
6 a	32.08	2.24, m	31.99	2.24, m
b	-	1.35, m	-	1.35, m
7	30.1	3.16, m	30.6	3.32, m
8	122.0	-	130.3	-
9	147.9	-	136.9	-
10	138.8	-	149.2	-
11	125.7	-	116.7	-
12	134.6	-	135.6	-
13	136.2	-	135.2	-
14	130.7	4.92, d (9.4)	130.5	
15	128.7	-	128.9	-
16	25.4	1.64, s	25.4	1.65, s
17	17.6	1.74, s	17.6	1.74, s
18	19.73	1.02, d (6.0)	19.77	1.02, d (6.0)
19	22.2	1.36, d (6.6)	23.8	1.41, d (6.6)
20	13.6	2.33, s	12.3	2.24, s
21	161.7	-	161.1	-
C21-Oxazole appendage (AA derived)	-	-	-	-
22	26.49	4.26, s	26.40	4.26, s
23	130.2	-	130.2	-
24	-	8.57 [b], br	-	8.57 [b], br
25	134.5	8.50 [b], br s	134.5	8.50 [b], br s
26	-	7.82 [b], br	-	7.82 [b], br
27	117.3	7.01, br s	117.5	7.01, br s

[a] CDCl3, 600 MHz (^{13}C: 150 MHz); assigned by HSQC, HMBC & COSY. [b] interchangeable assignments. Note: The NMR data was acquired on material purified by HPLC ($MeOH:H_2O:HCO_2H$) and thus the imidazole moiety was protonated. The chemical shifts are sensitive to changes in pH.

Figure S6. ^{1}H-NMR (600 MHz, $CDCl_3$) spectrum of **7a/7b** mixture.

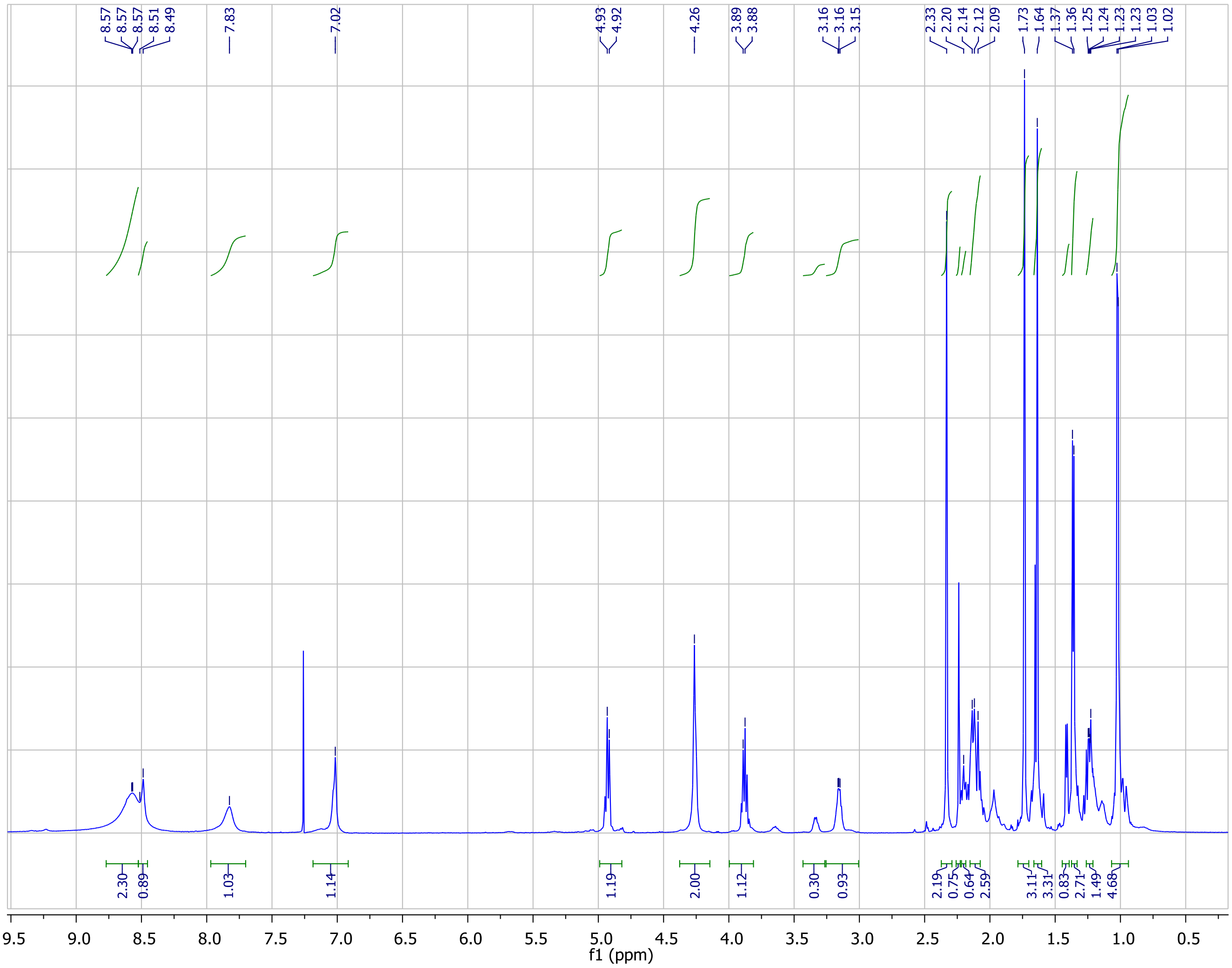

Figure S7. ^{13}C-NMR (150 MHz, $CDCl_3$) spectrum of **7a/7b** mixture.

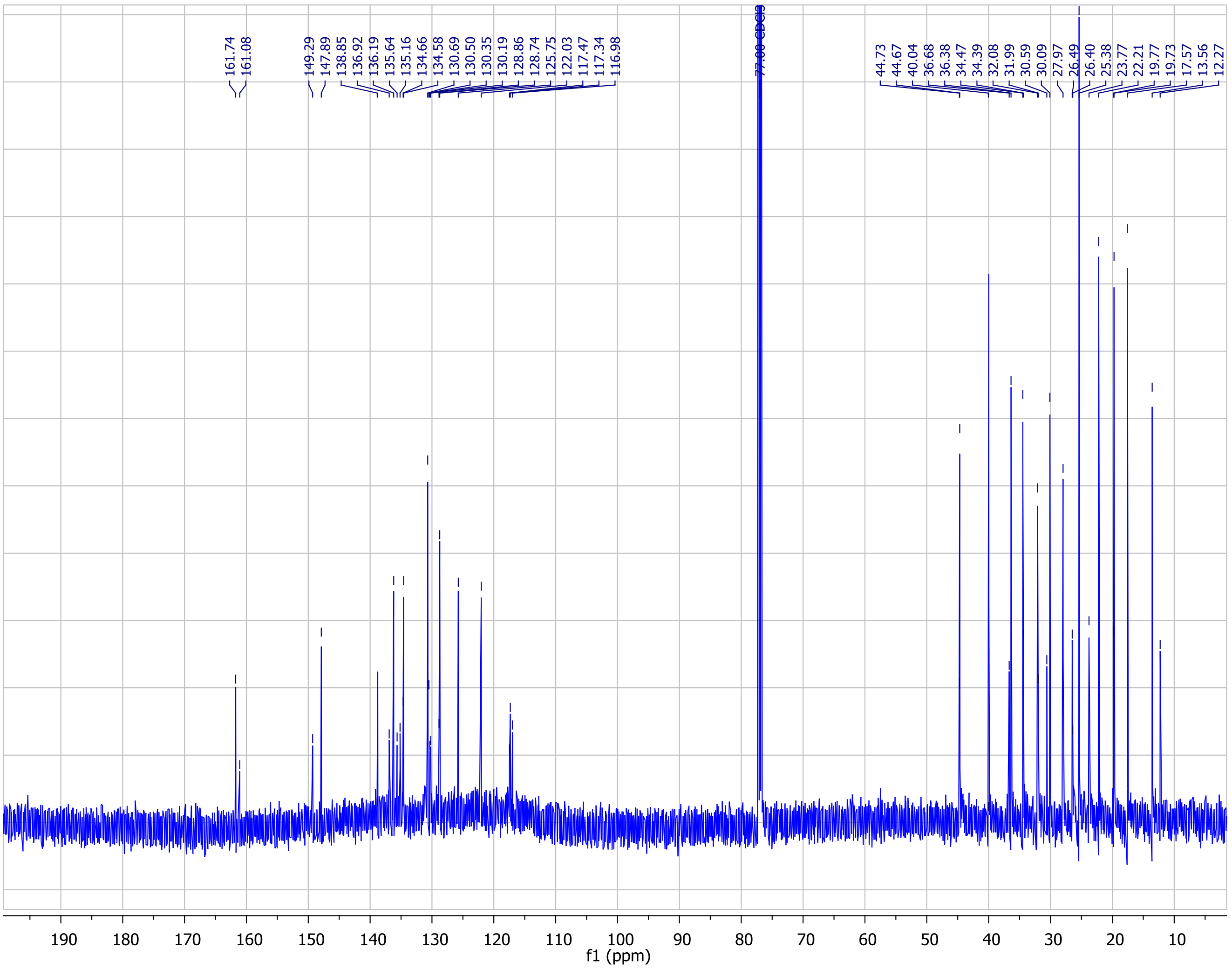

Figure S8. HSQC spectrum (600 MHz, $CDCl_3$) of **7a/7b** mixture.

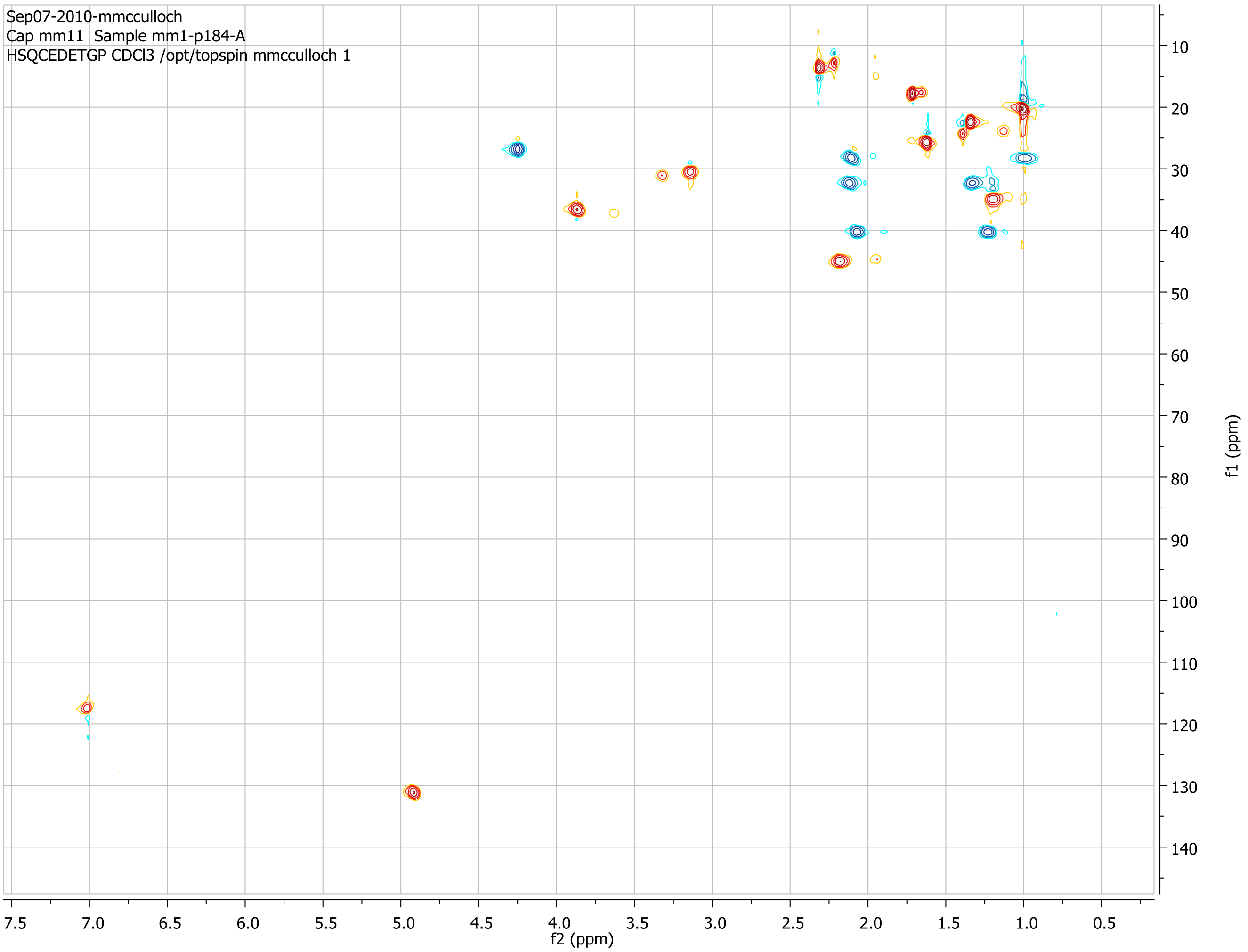

Figure S9. MS (top) and MSMS spectra (bottom) of **7a**/**7b**.

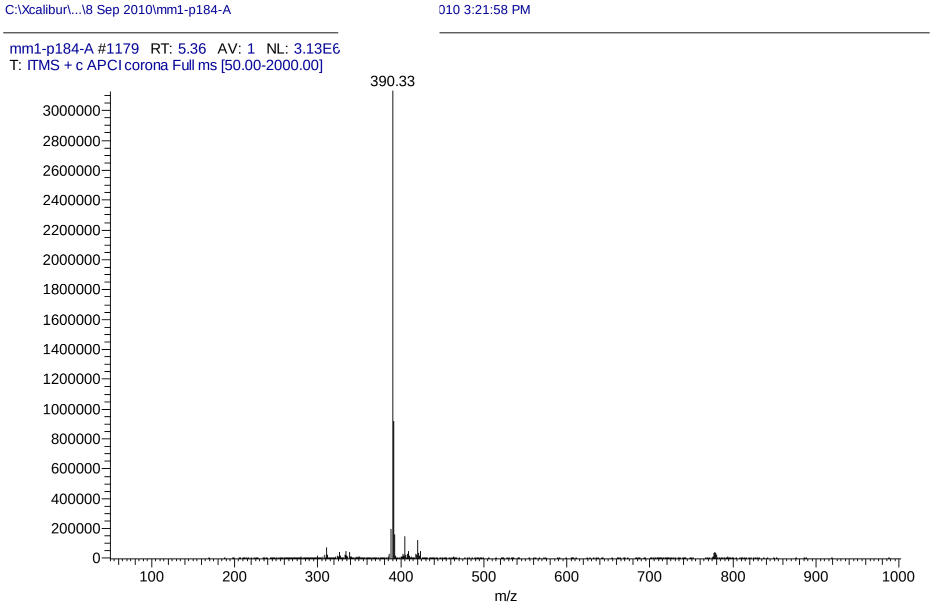

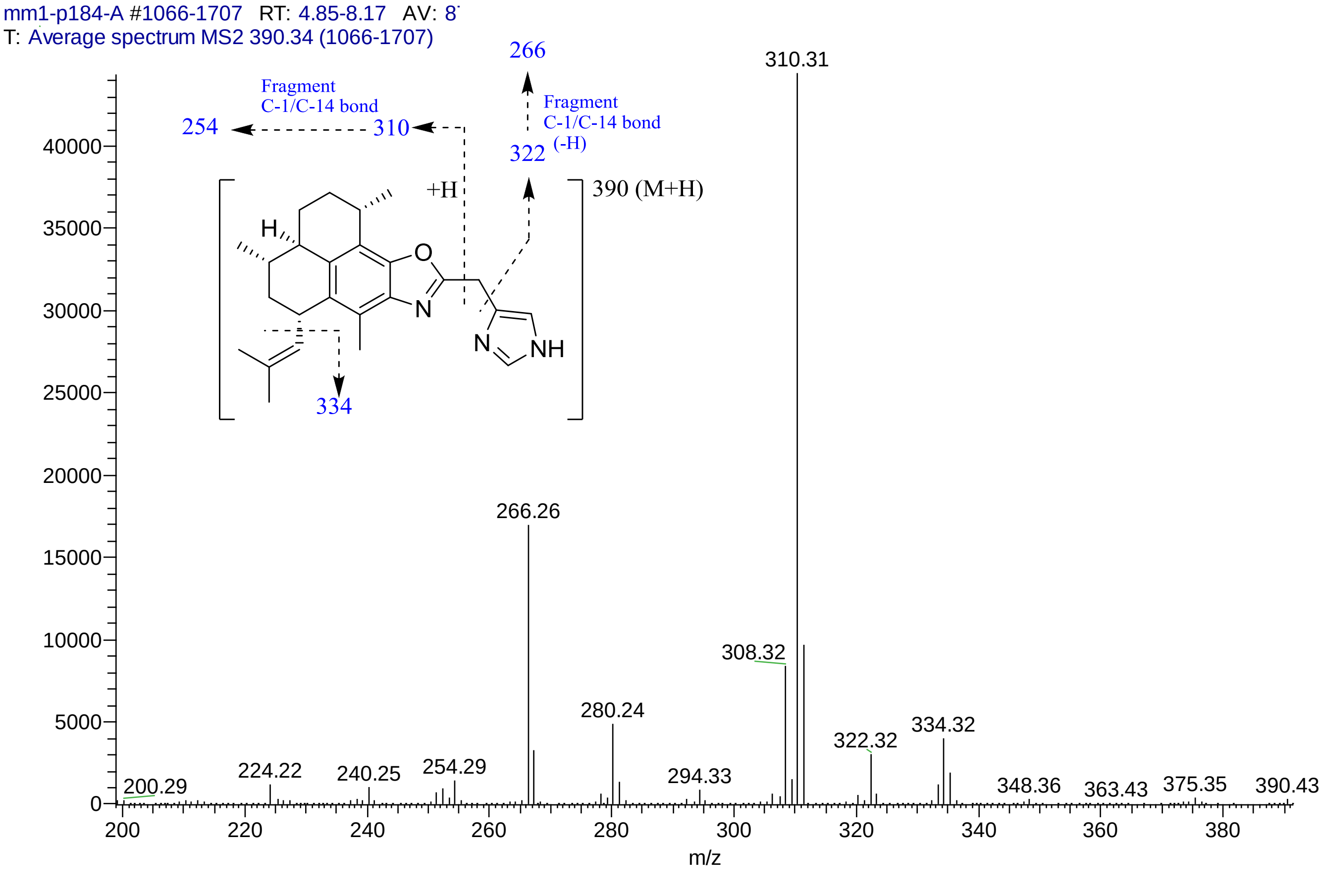

Figure S10. Analytical UPLC Chromatogram of **7a/7b**. (**a**) UV trace 255 nm; (**b**) ELSD trace; (**c**) Base peak mass chromatogram; (**d**) Selected ion monitoring *m/z* 390 [M + H]; (**e**) Average mass spectrum (5.15–5.45 min).

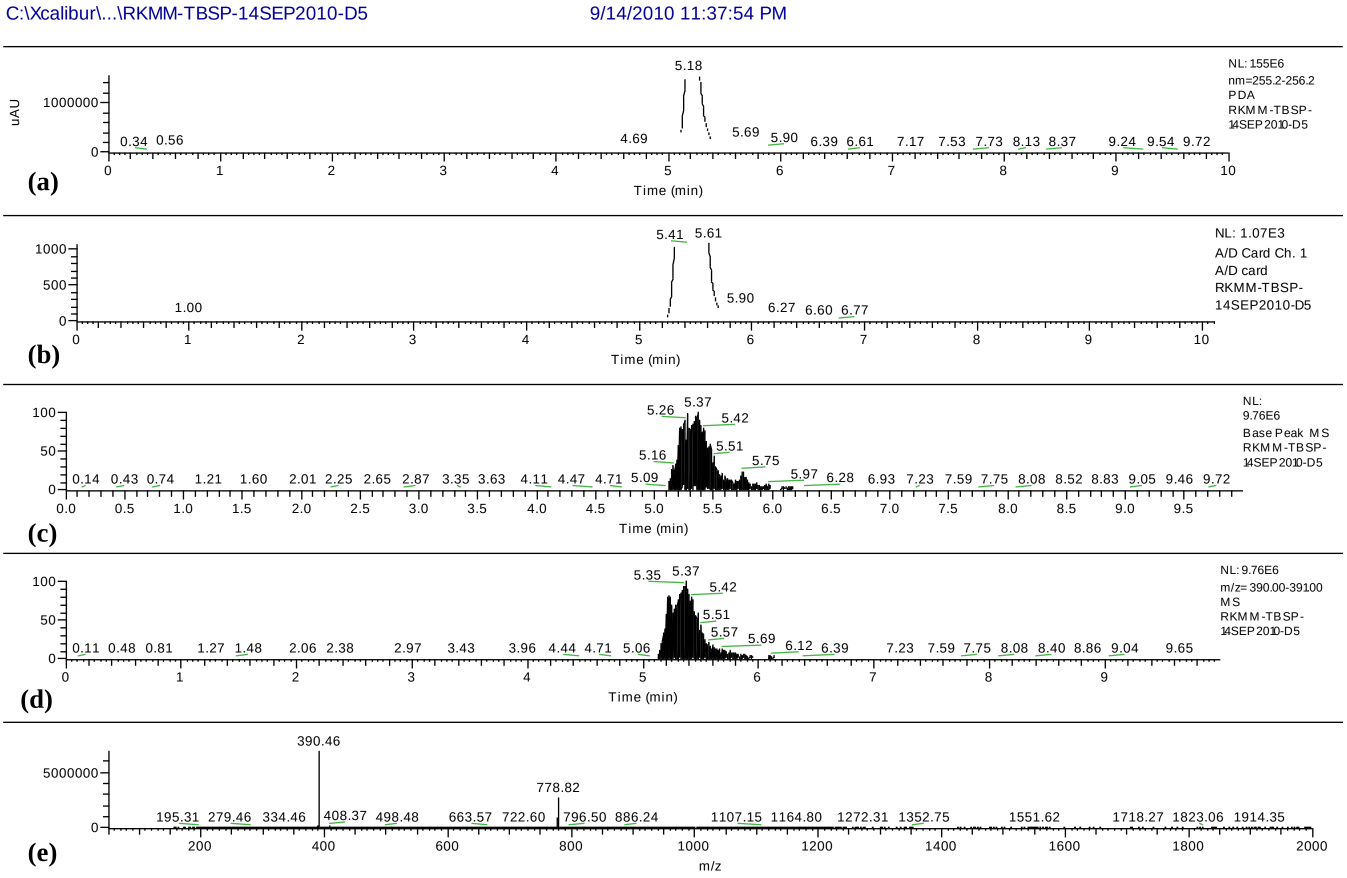

Figure S11. ^{1}H-NMR (600 MHz, $CDCl_3$) spectrum of **8**.

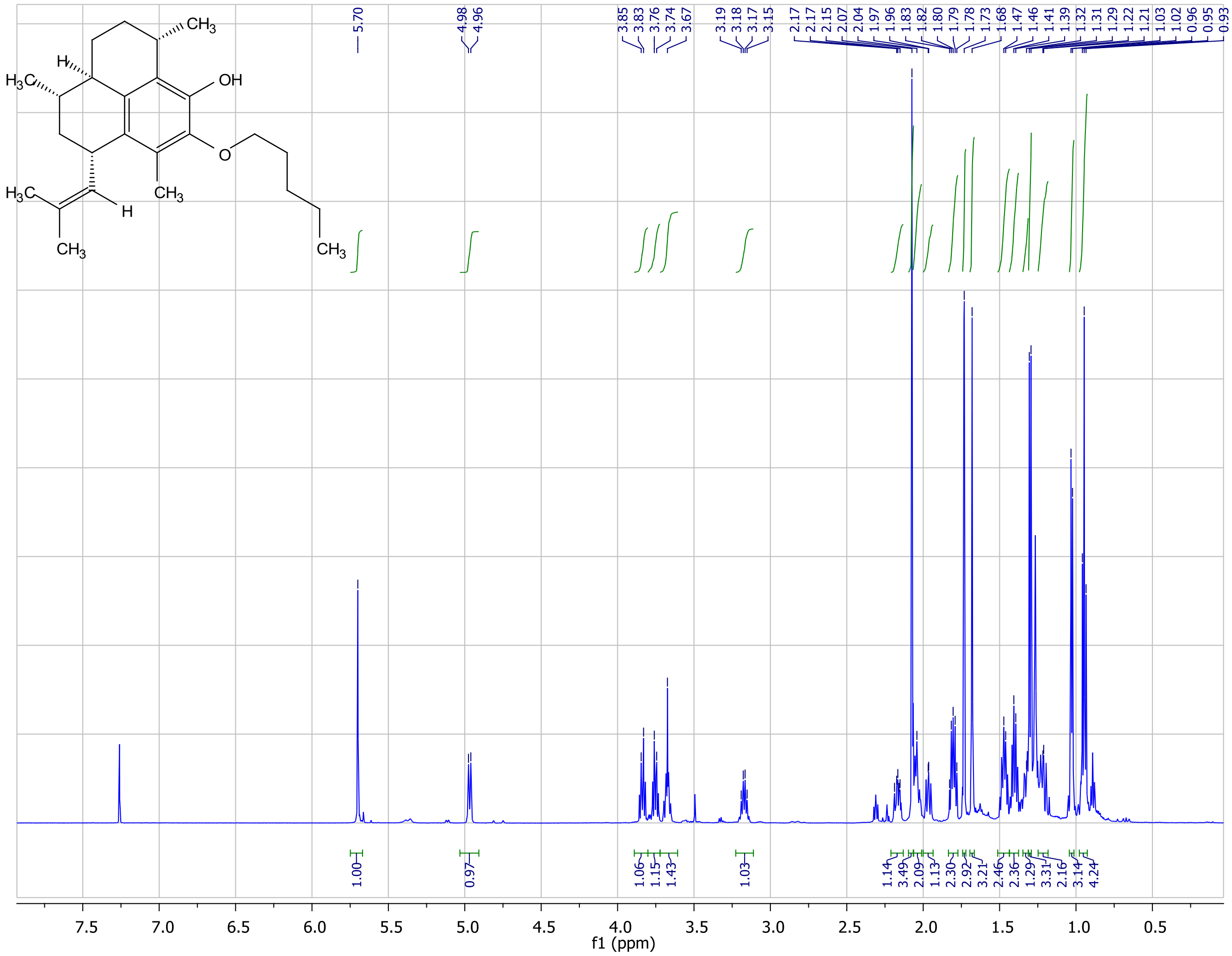

Figure S12. ^{13}C-NMR (150 MHz, $CDCl_3$) spectrum of **8**.

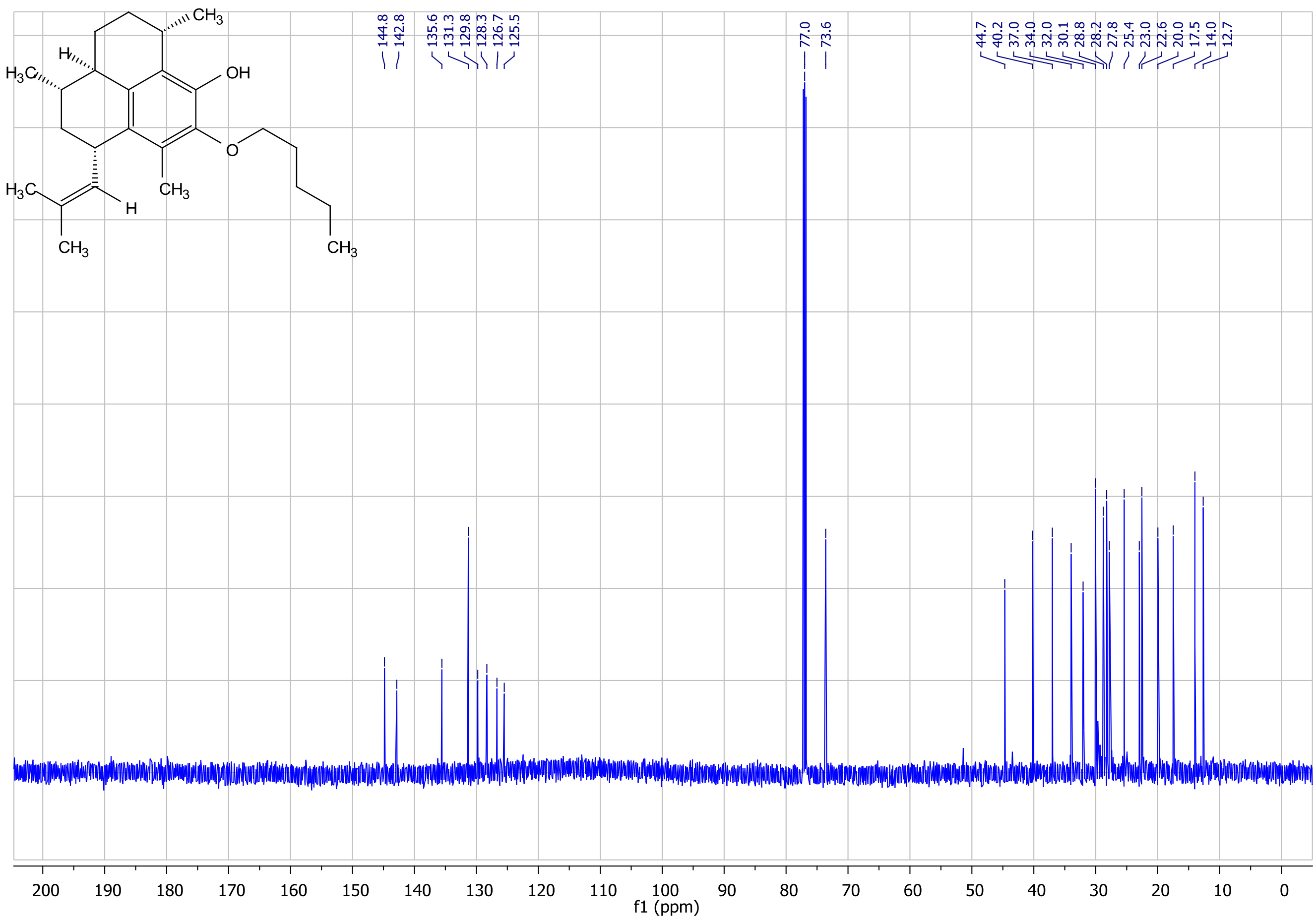

Figure S13. ^{1}H-NMR (600 MHz, $CDCl_3$) spectrum of **10**.

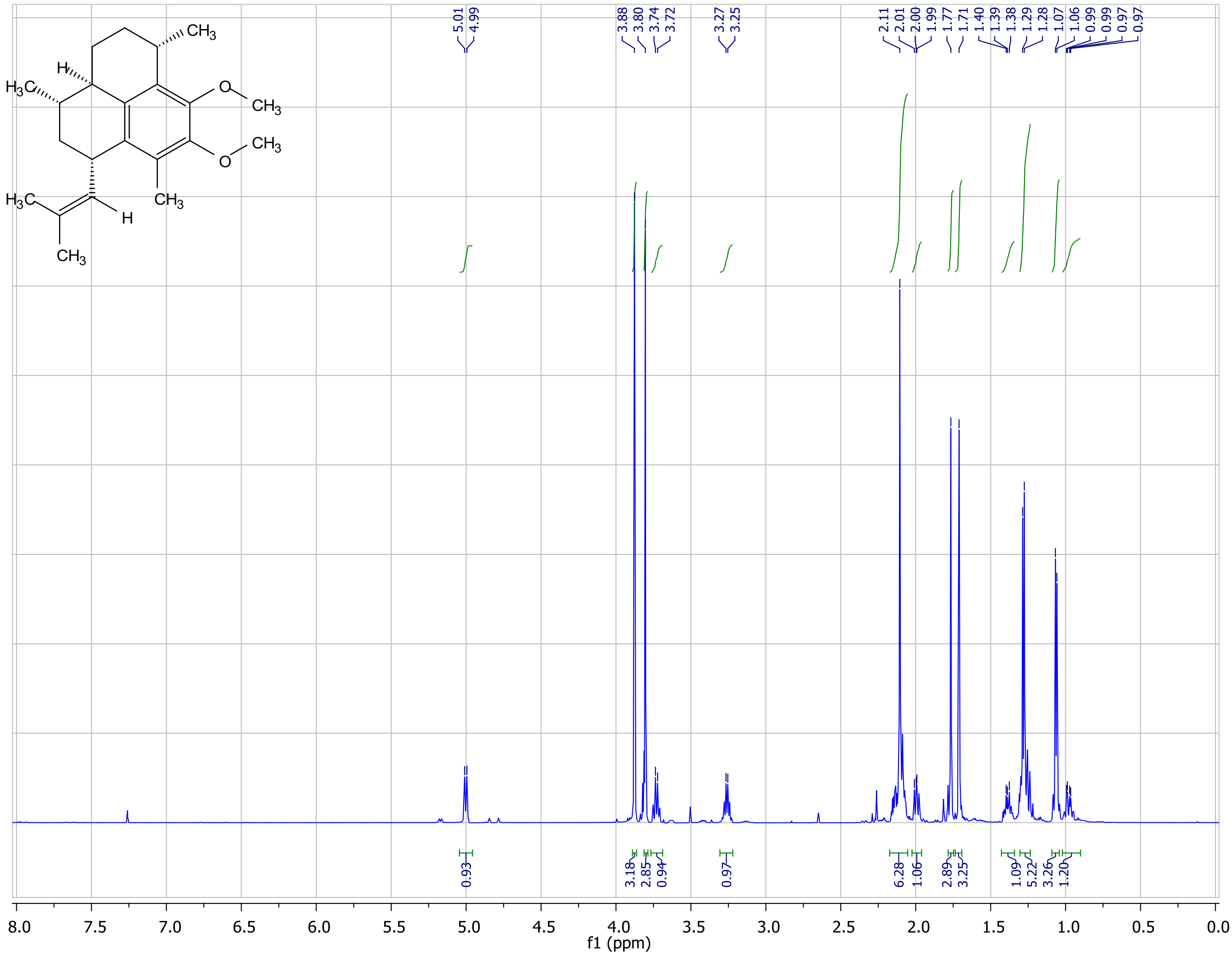

Figure S14. ^{13}C-NMR (150 MHz, $CDCl_3$) spectrum of **10**.

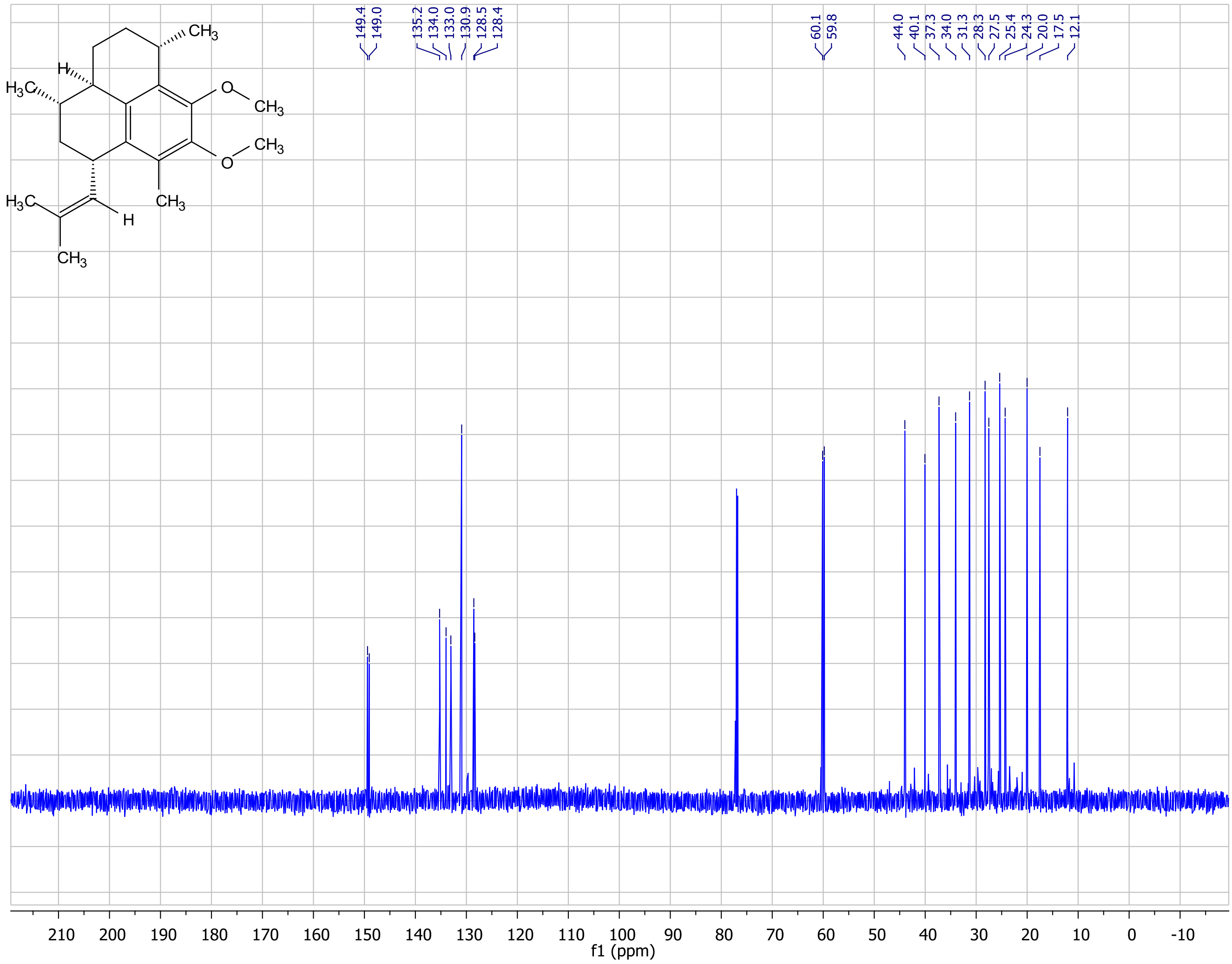

Figure S15. ^{1}H-NMR (600 MHz, $CDCl_3$) spectrum of **11**.

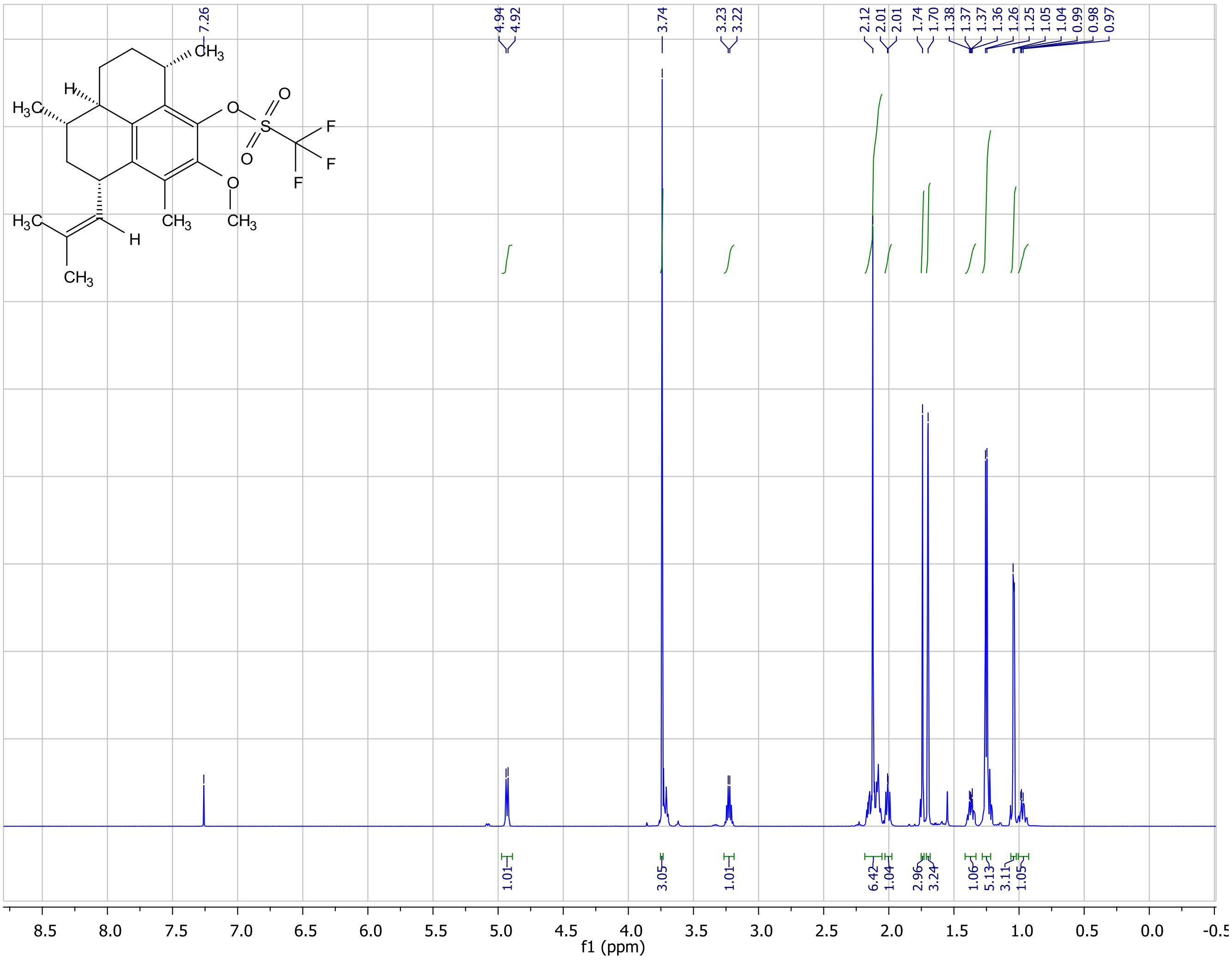

Figure S16. ^{13}C-NMR (150 MHz, $CDCl_3$) spectrum of **11**.

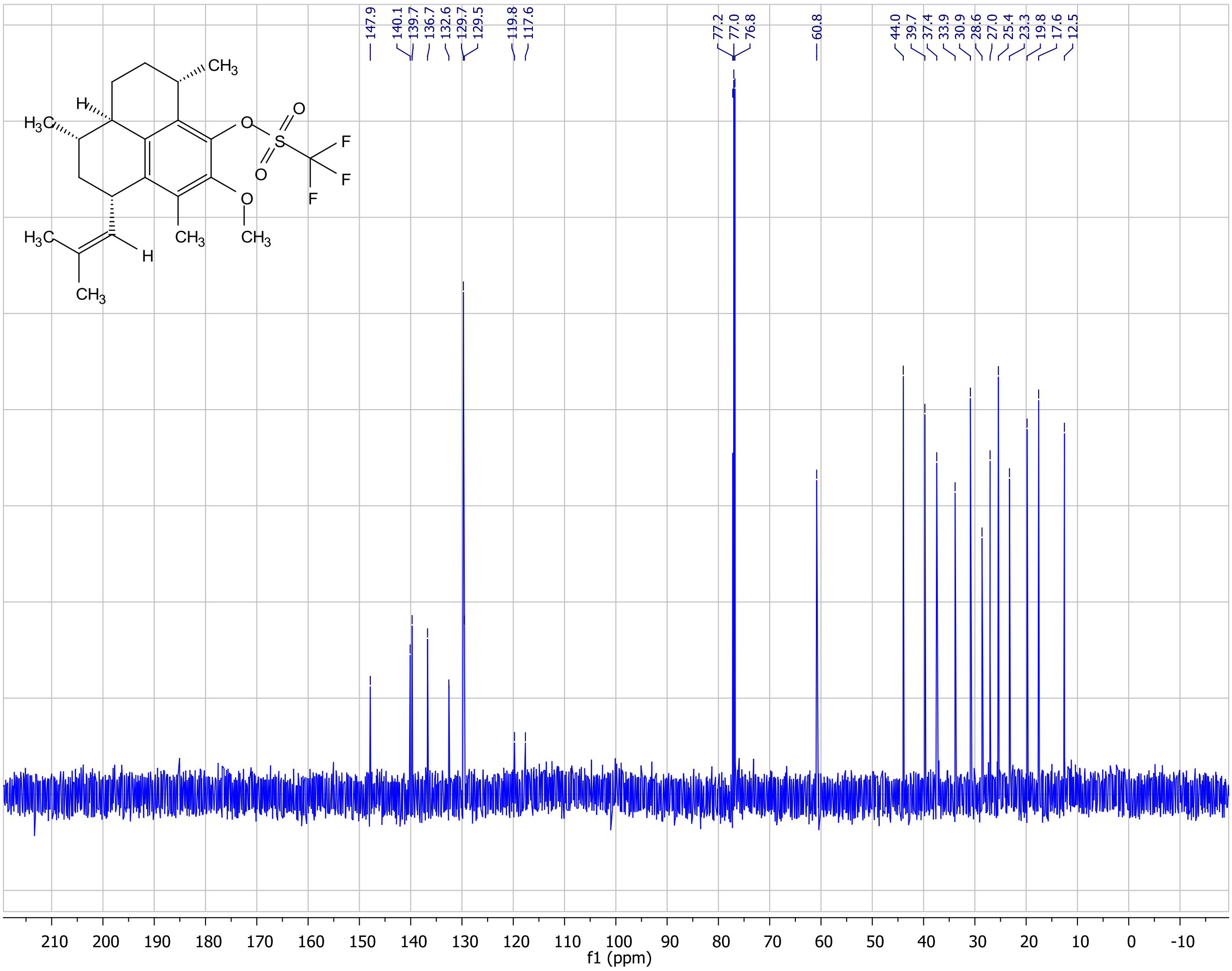

Figure S17. ^{1}H-NMR (600 MHz, $CDCl_3$) spectrum of **12**.

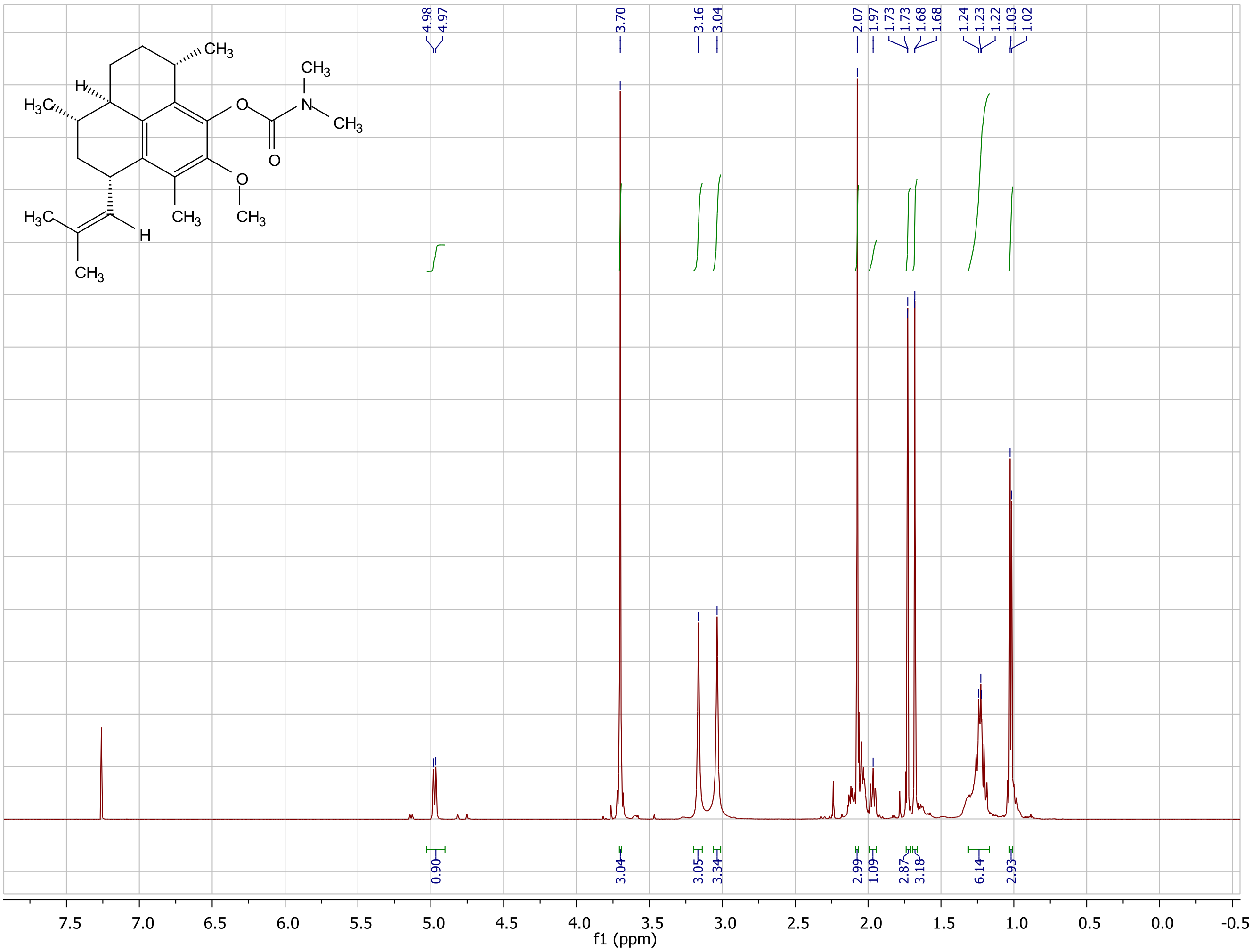

Figure S18. ^{13}C-NMR (150 MHz, $CDCl_3$) spectrum of **12**.

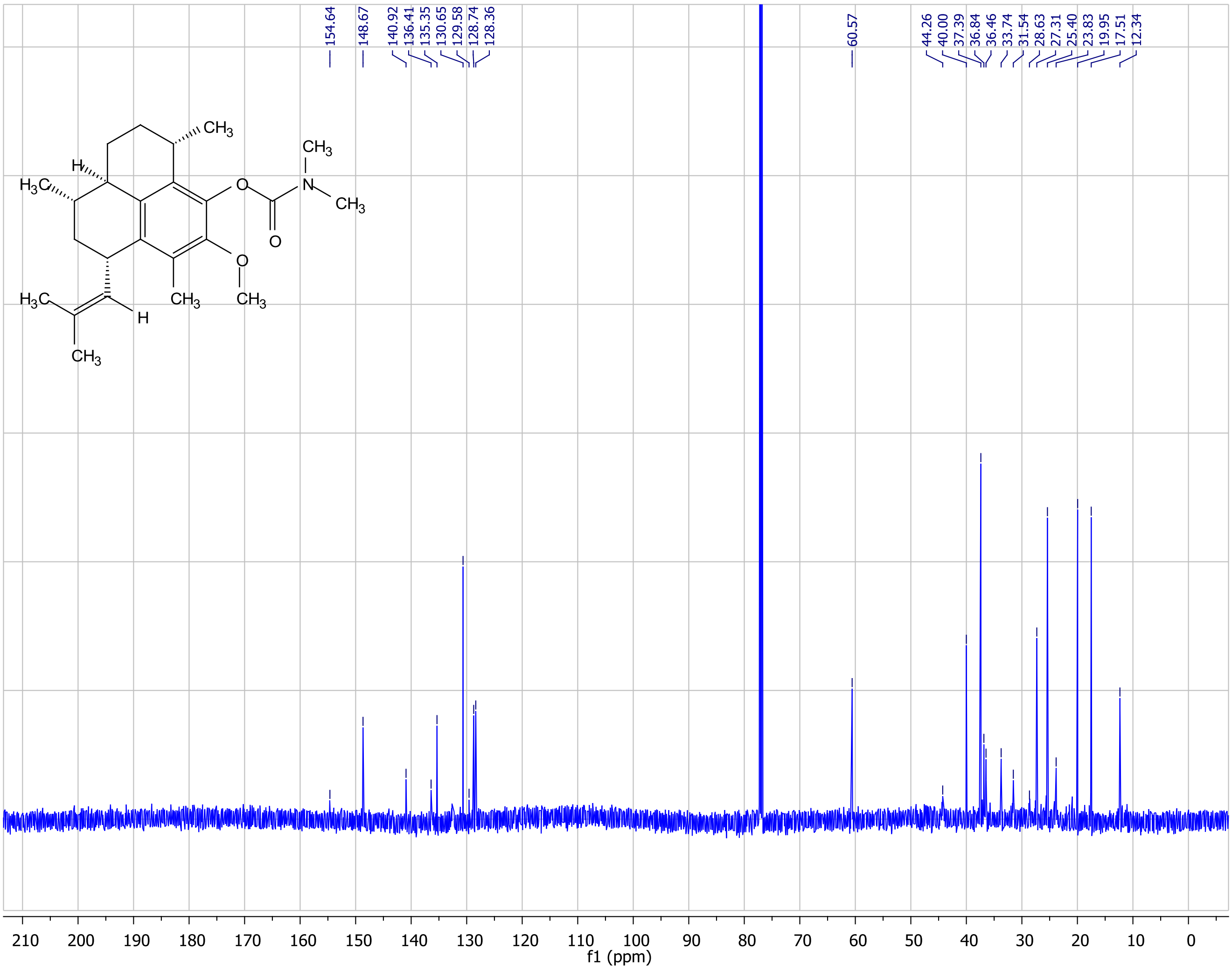

Figure S19. ^{1}H-NMR (600 MHz, $CDCl_3$) spectrum of **14**.

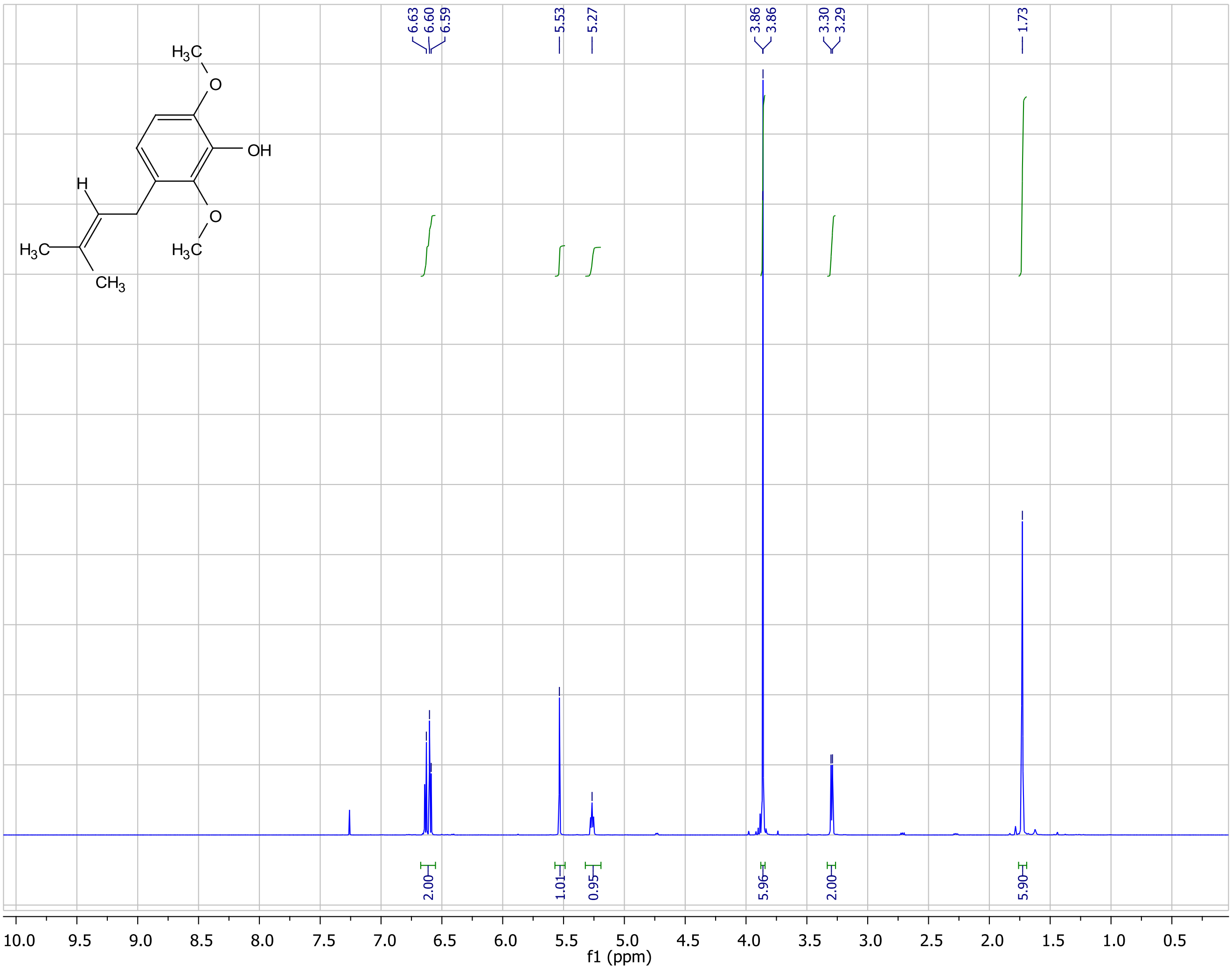

Figure S20. ^{13}C-NMR (150 MHz, $CDCl_3$) spectrum of **14**.

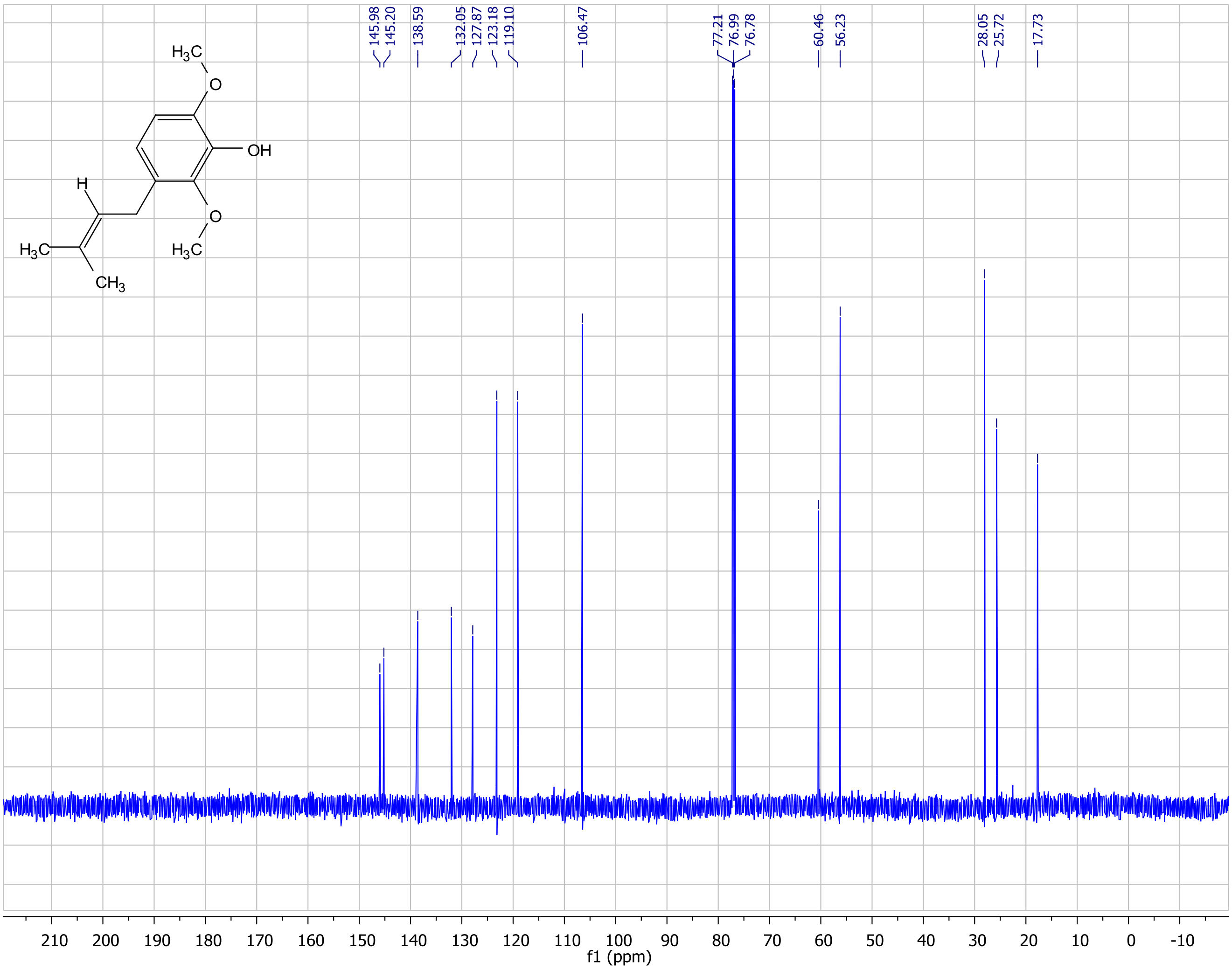

Figure S21. ^{1}H-NMR (600 MHz, $CDCl_3$) spectrum of **15**.

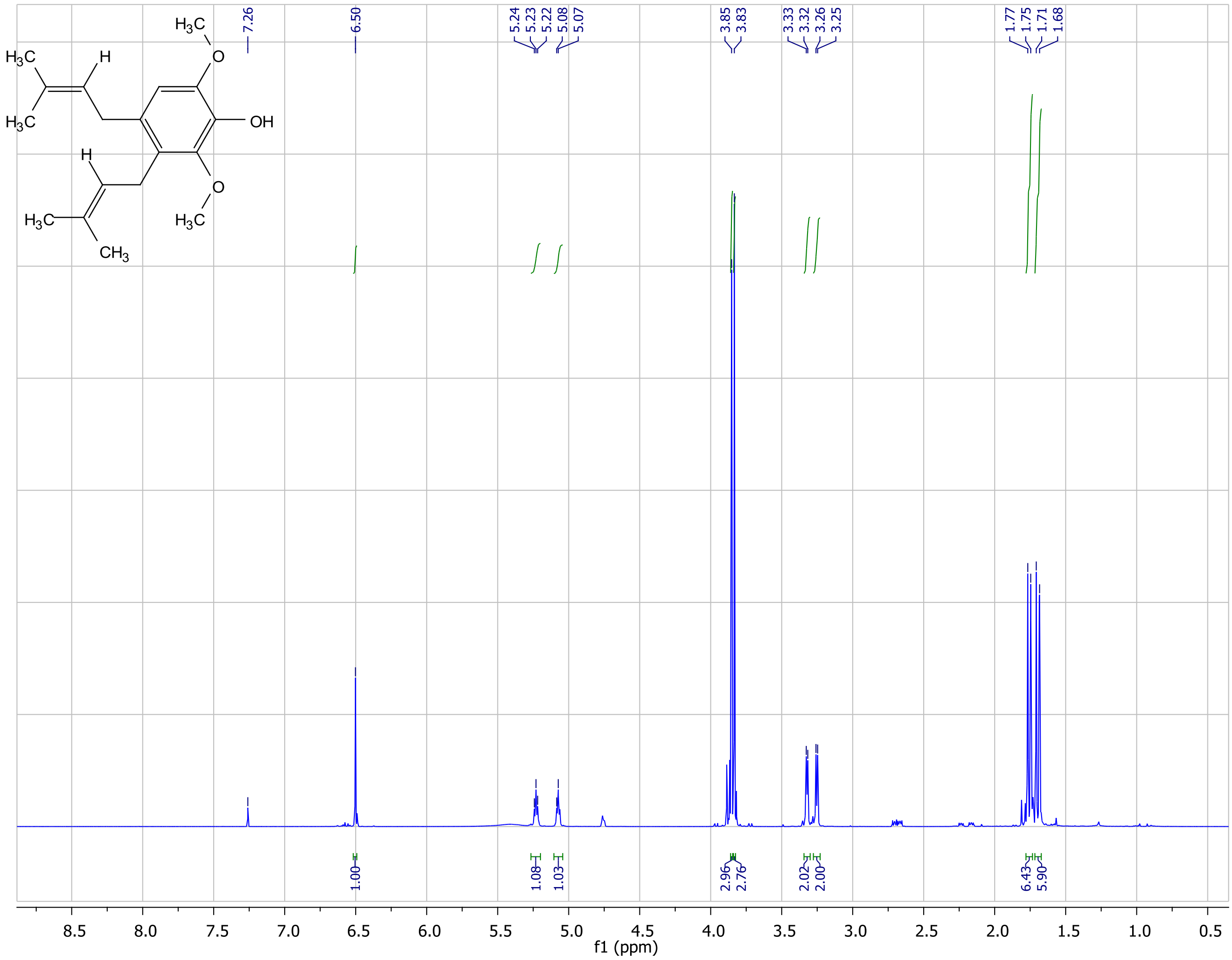

Figure S22. ^{13}C-NMR (150 MHz, $CDCl_3$) spectrum of **15**.

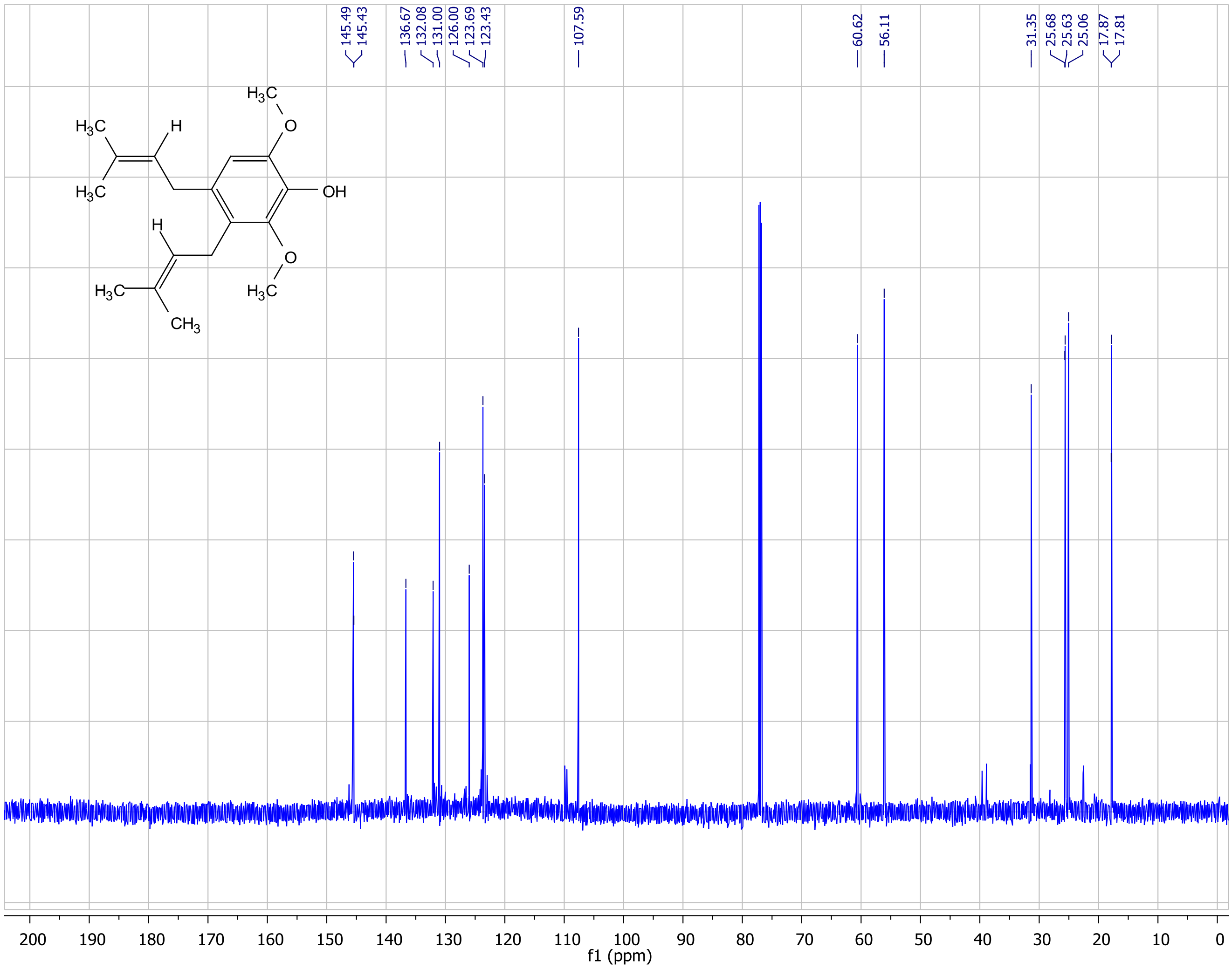

Figure S23. ^{1}H-NMR (600 MHz, $CDCl_3$) spectrum of **16**.

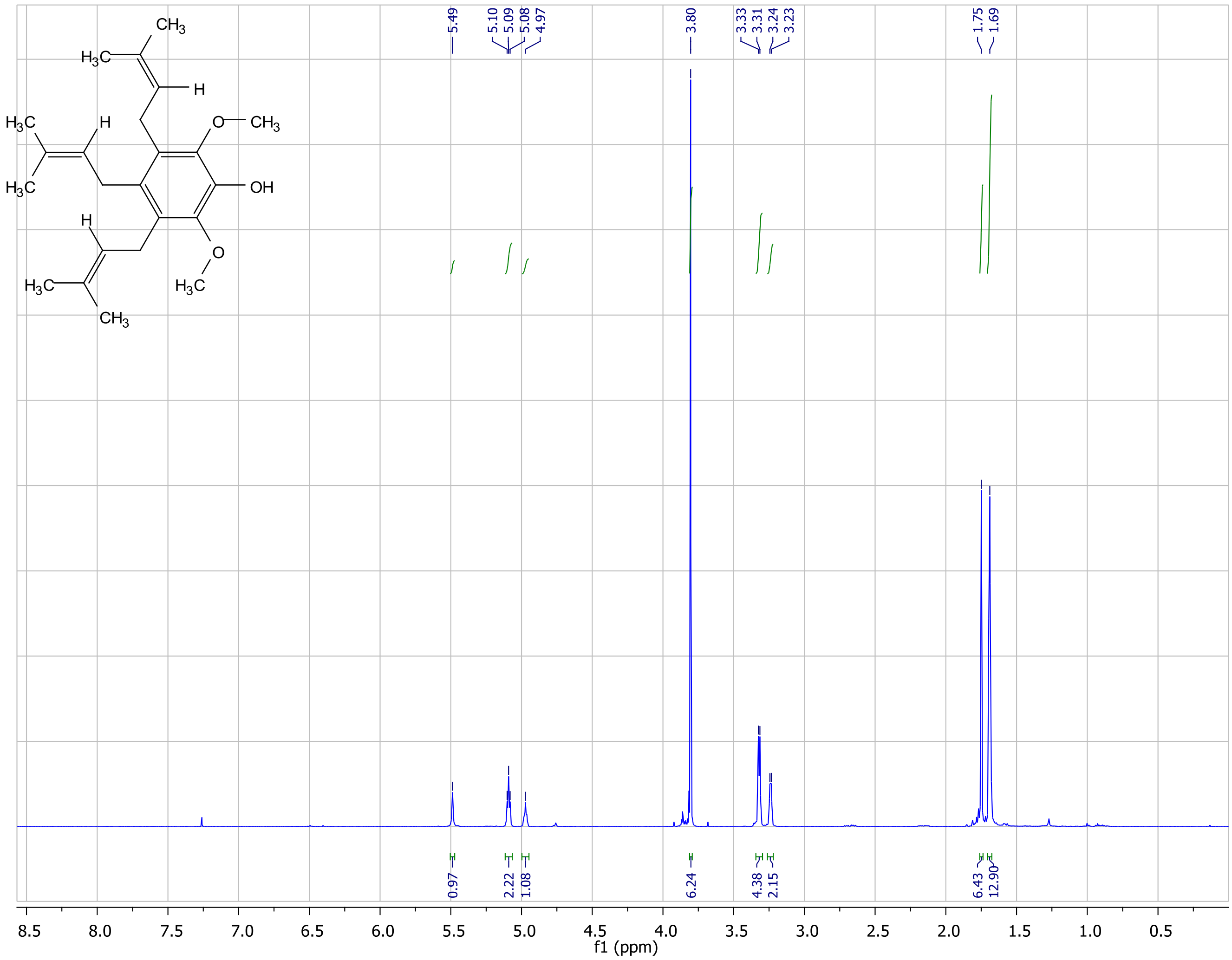

Figure S24. ^{13}C-NMR (150 MHz, $CDCl_3$) spectrum of **16**.

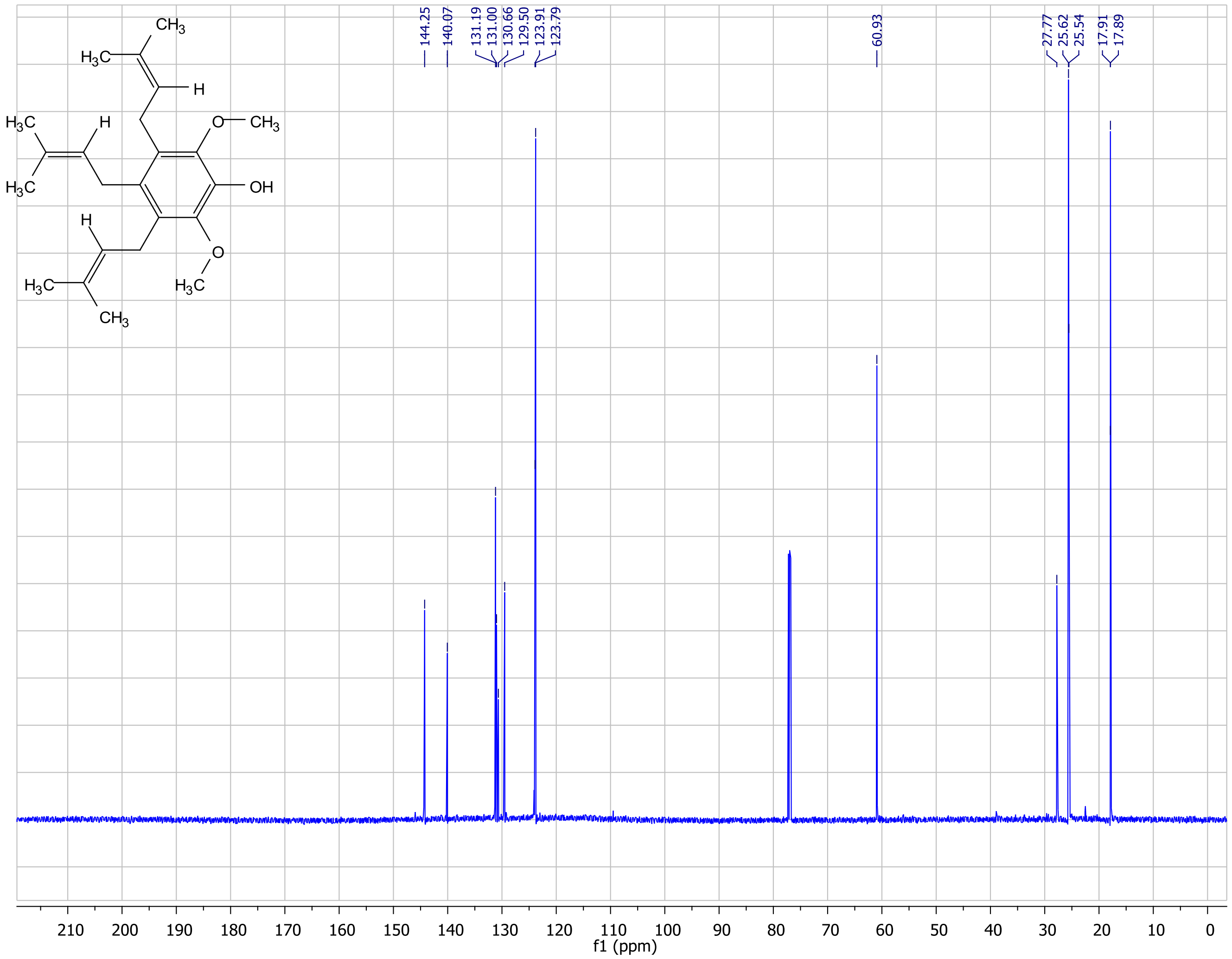

Figure S25. ^{1}H-NMR (600 MHz, C_6D_6) spectrum of **17**.

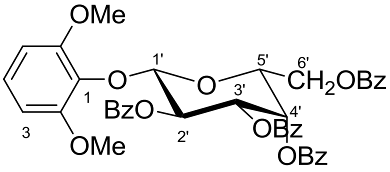

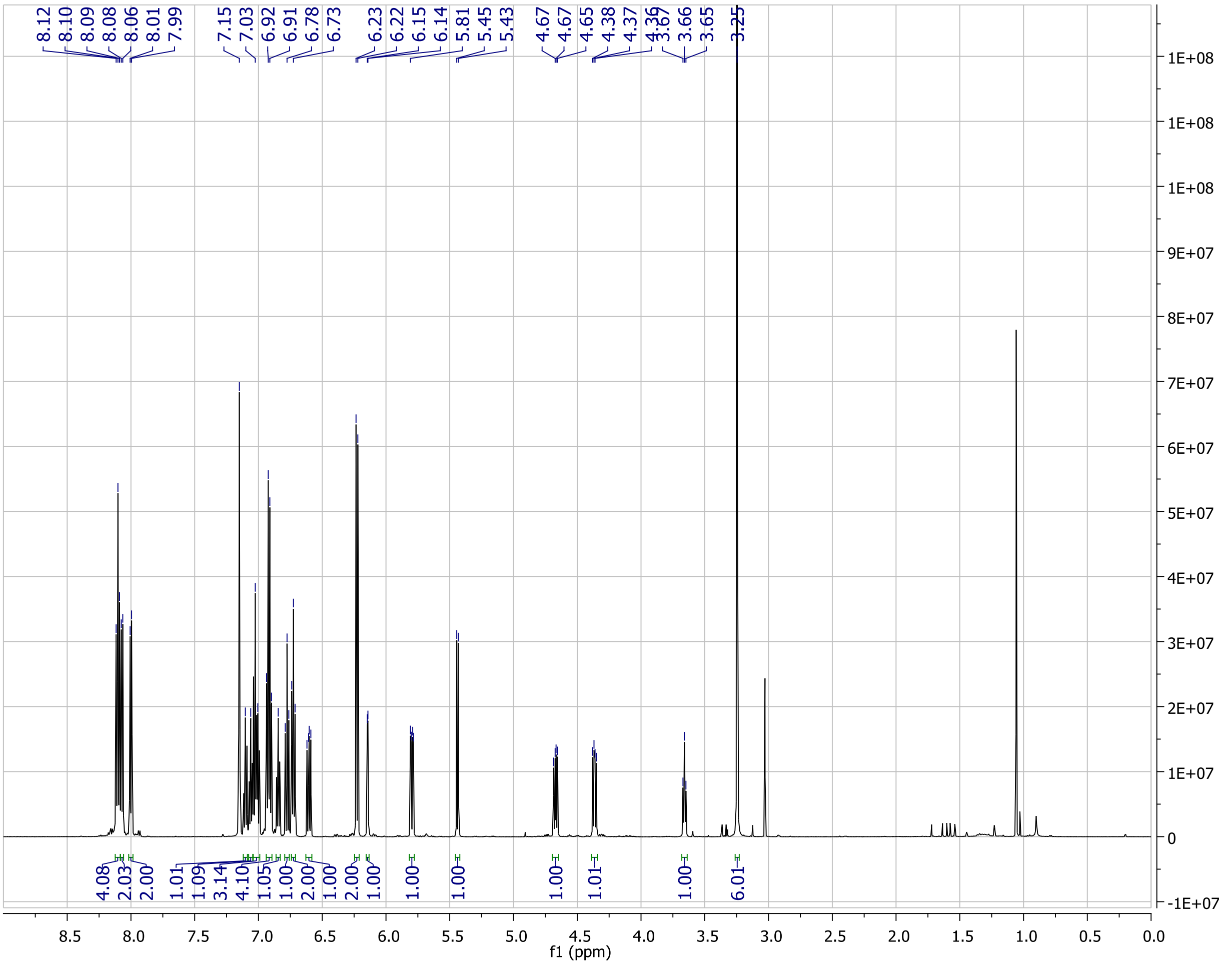

Figure S26. ^{13}C-NMR (600 MHz, C_6D_6) spectrum of **17**.

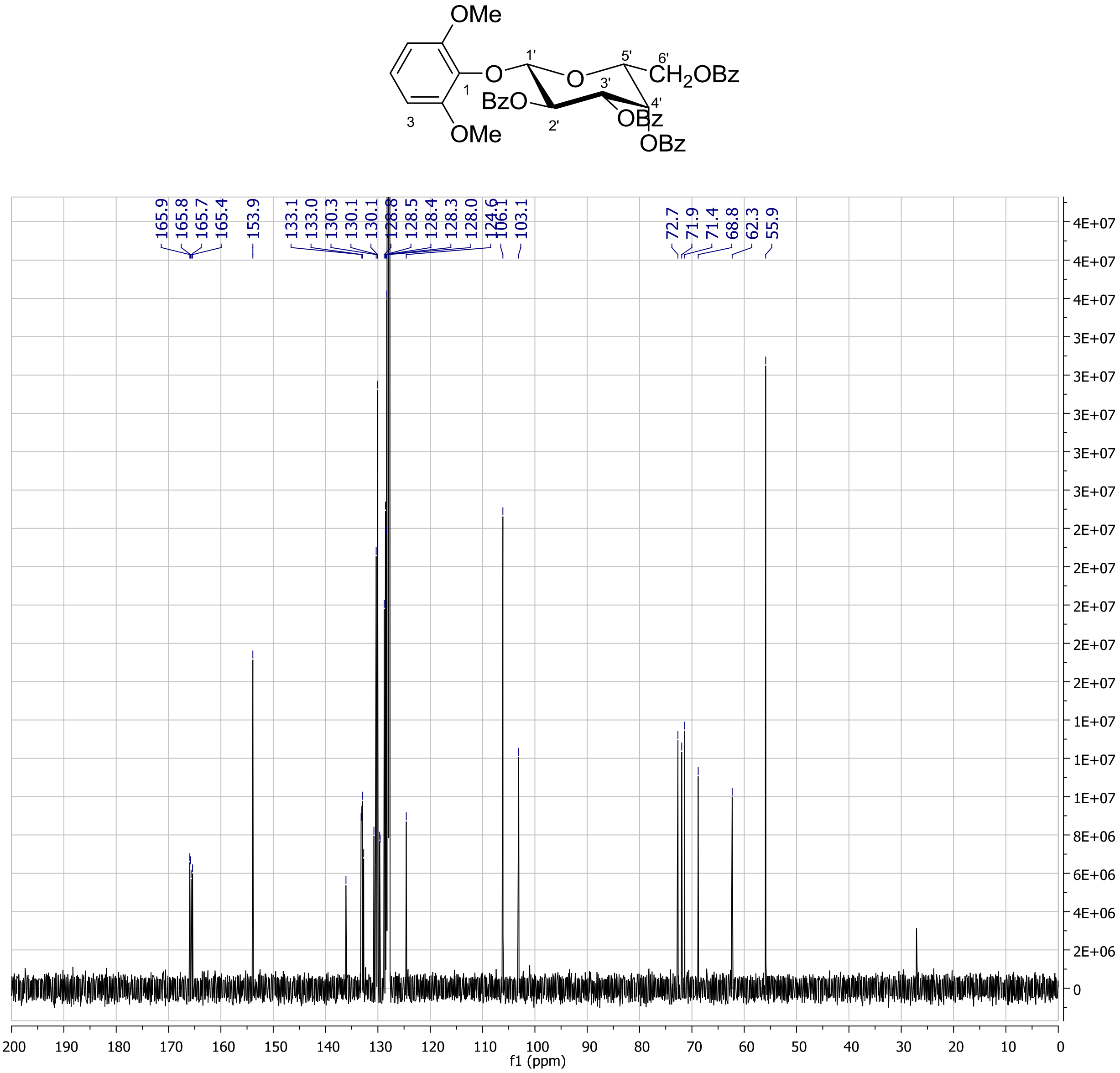

Figure S27. ^{1}H-NMR (600 MHz, $(CD_3)_2SO$) spectrum of **18**.

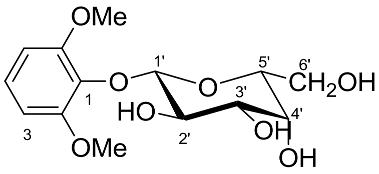

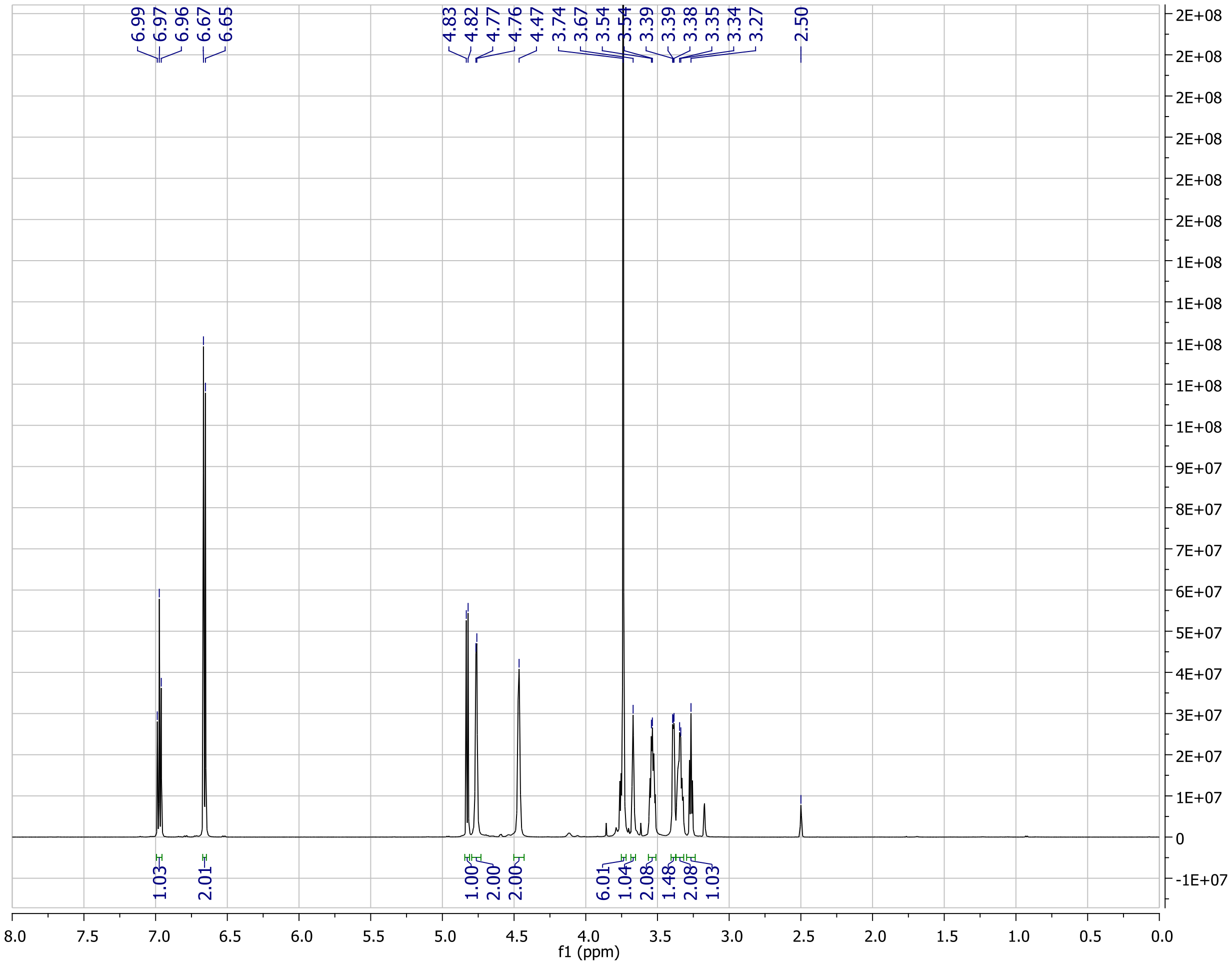

Figure S28. ^{13}C-NMR (600 MHz, $(CD_3)_2SO$) spectrum of **18**.

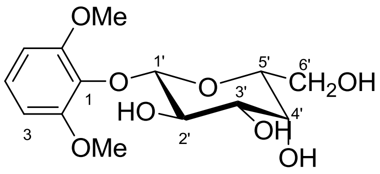

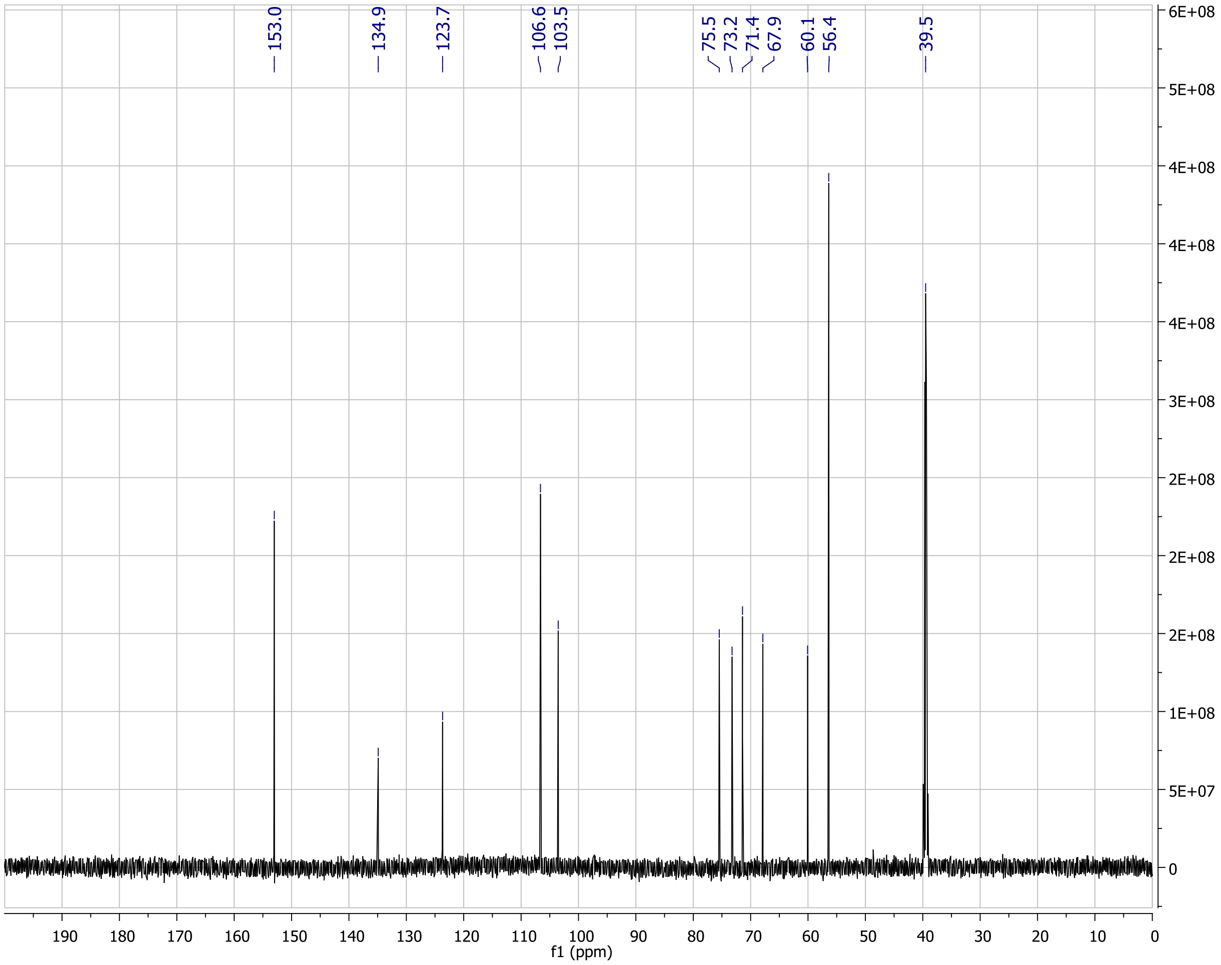

Figure S29. ^{1}H-NMR (600 MHz, CD_3OD) spectrum of **19**.

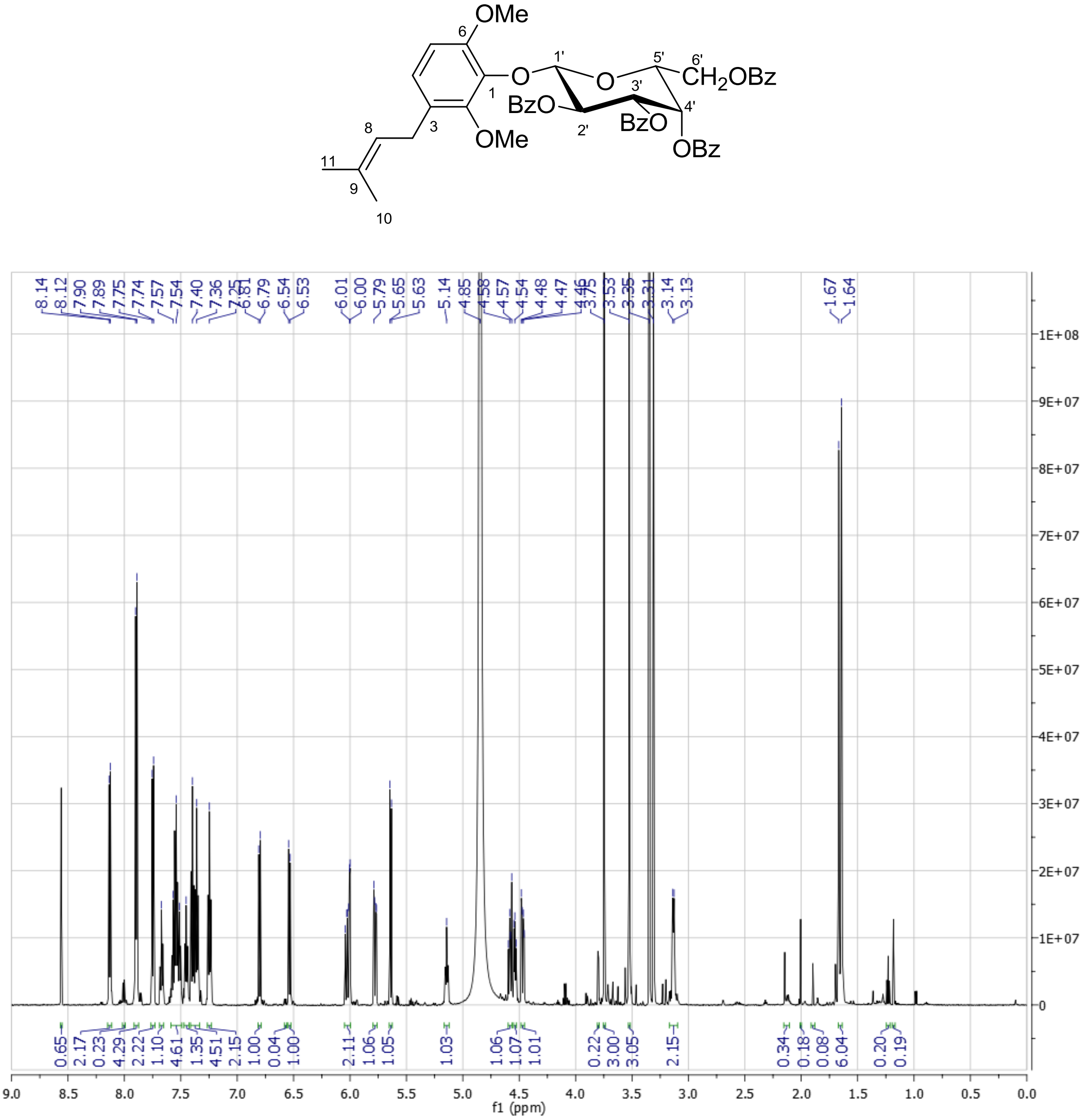

Note: some impurities are present: residual solvent CH_3OD at δ 3.35 and H_2O at δ 4.85. Baseline impurities appear to be glycosylated analogue(s) of **19**. Further HPLC purification of **19** was unsuccessful, however following deprotection compound **20** was purified and characterized.

Figure S30. ^{13}C-NMR (600 MHz, CD_3OD) spectrum of **19**.

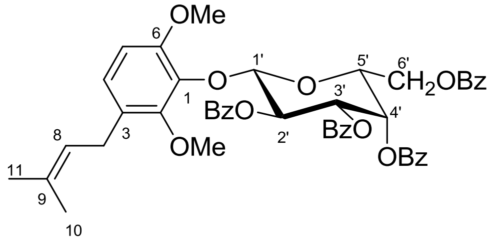

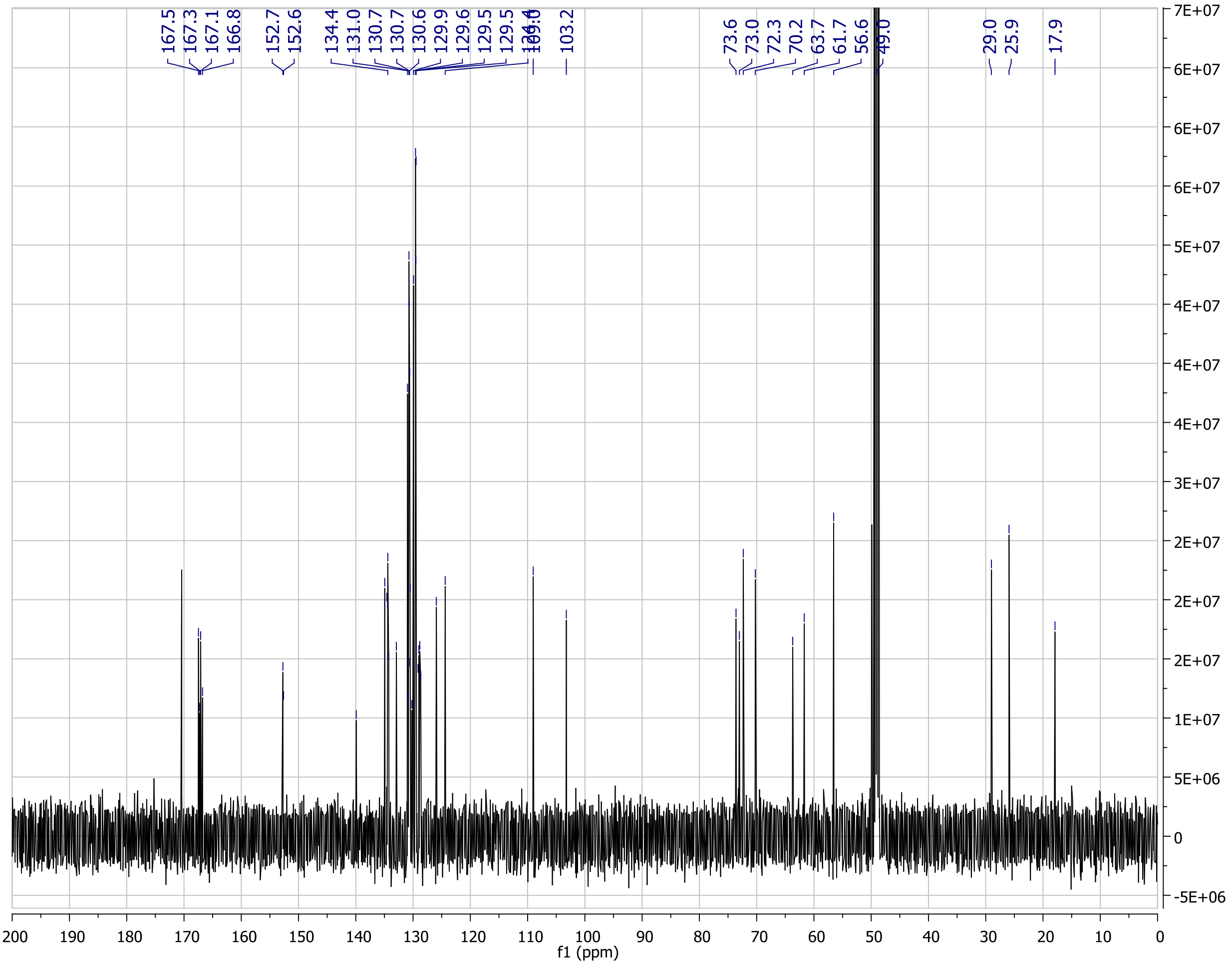

Figure S31. ^{1}H-NMR (600 MHz, $(CD_3)_2SO$) spectrum of **20**.

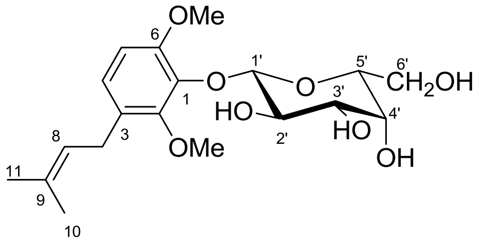

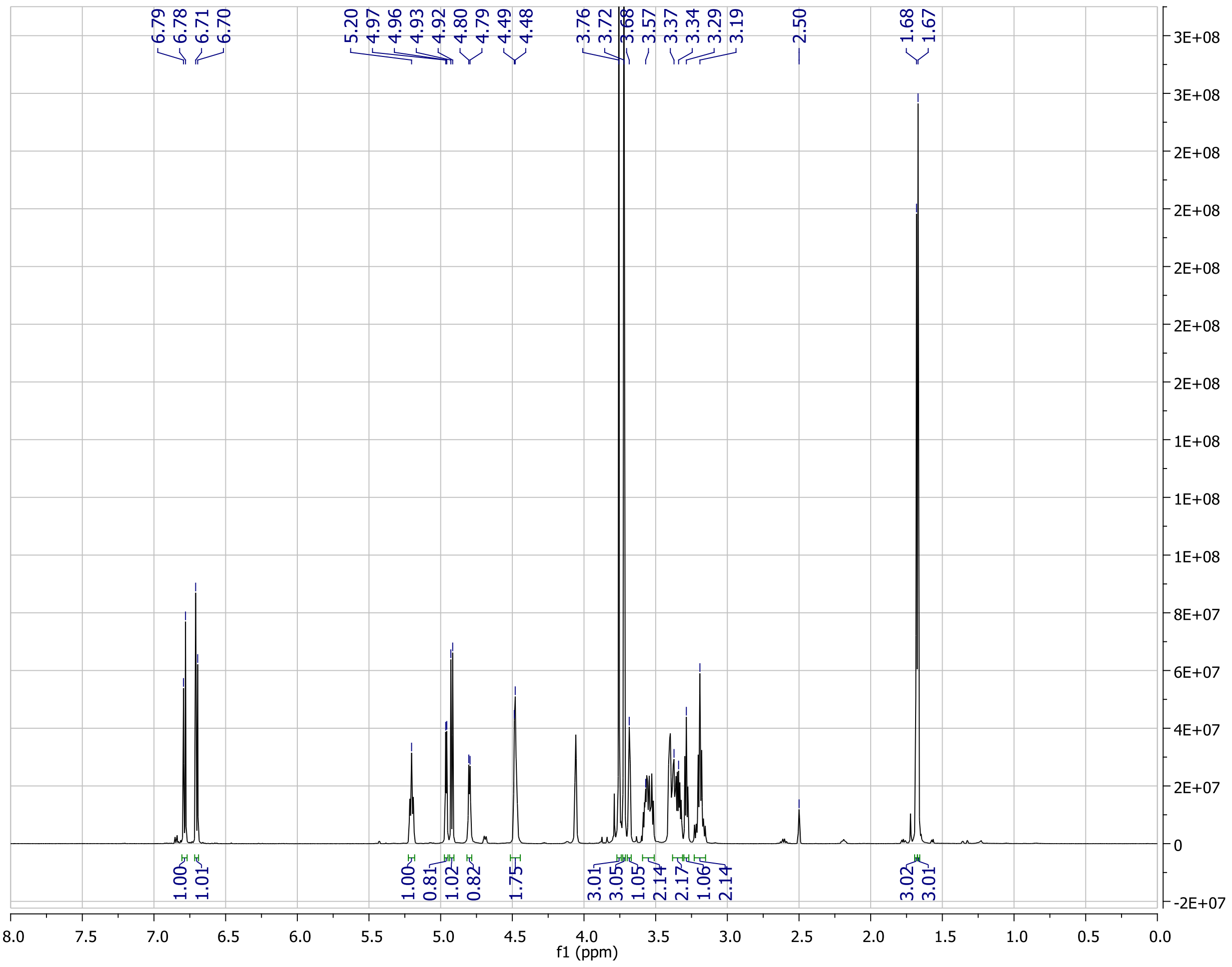

Figure S32. ^{13}C-NMR (600 MHz, $(CD_3)_2SO$) spectrum of **20**.

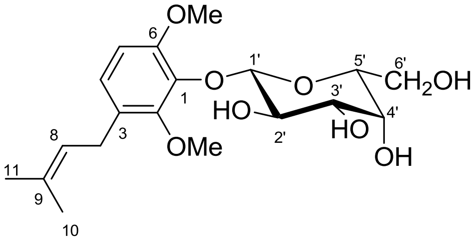

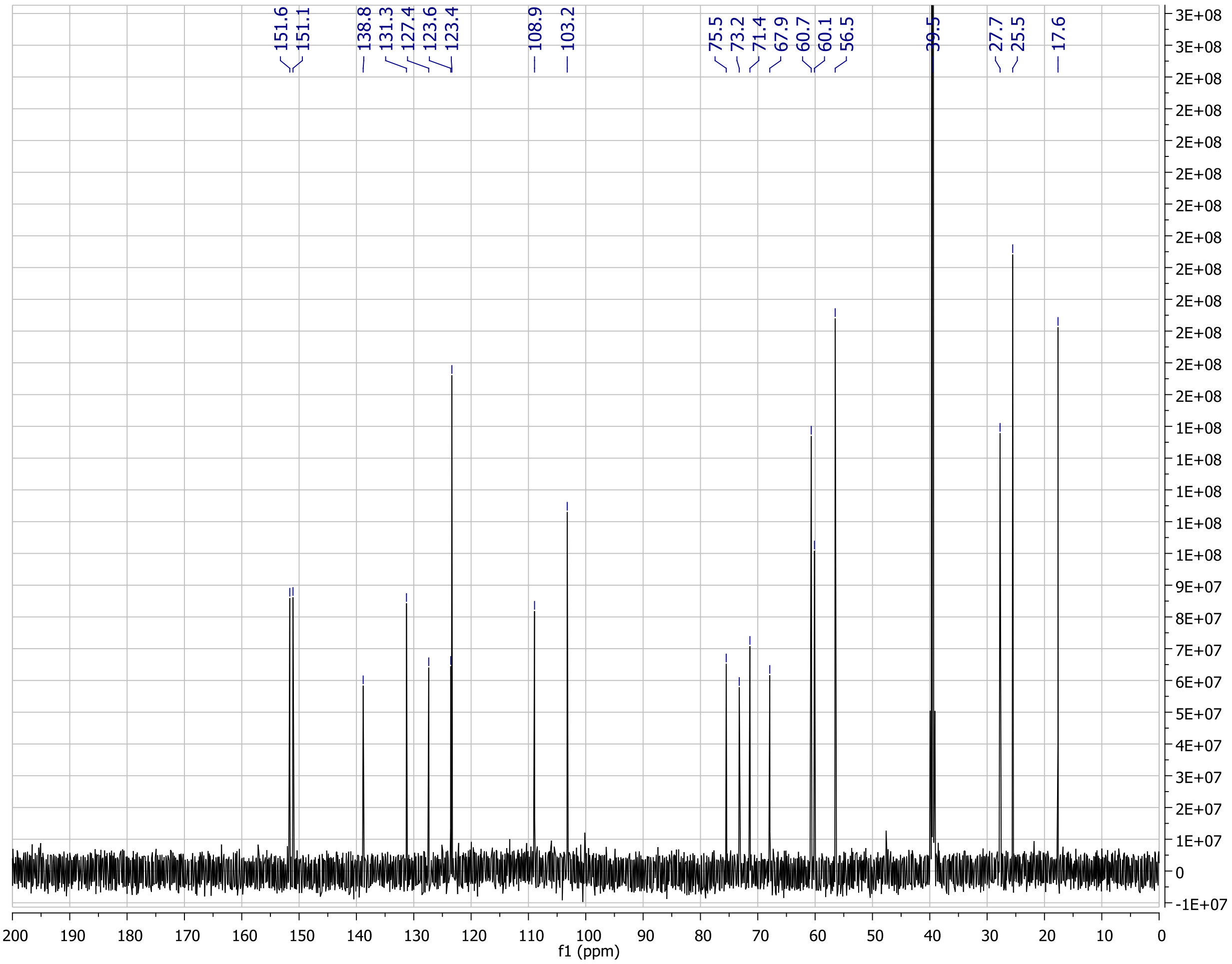

Scheme S1. Syntheses of 2,3,4,6-tetra-*O*-benzoyl-β-D-galactopyranosyl trichloro acetimidate (**21**). Reagents and conditions: (**a**) BzCl (7.0 equiv.), Pyr; (**b**) HBr (3.0 equiv.), MeOH (2.0 equiv.), AcOH, 0 ℃ → r.t., 36% over two steps; (**c**) Ag_2CO_3 (1.2 equiv.), acetone:H_2O (19:1), 85%; (**d**) CCl_3CN (10 equiv.), K_2CO_3 (1.2 equiv.), DCM, r.t. 36%.

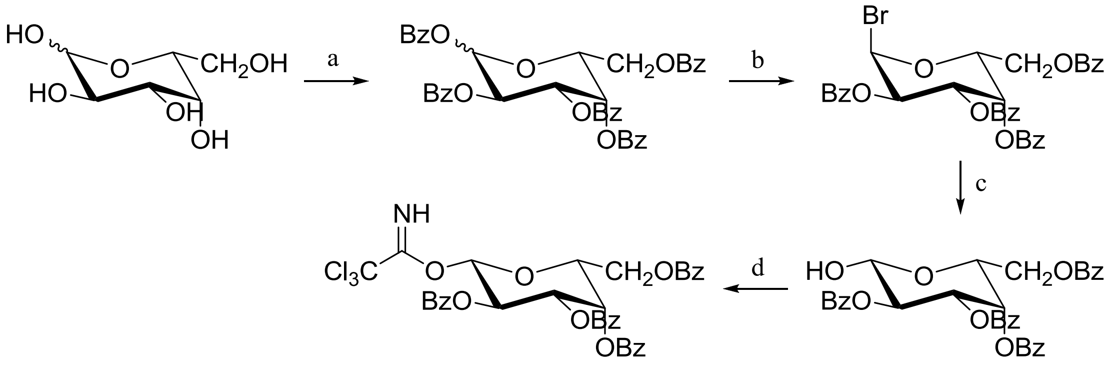

Supplement: Supplementary File 1: — PDF-Document (PDF, 2611 KB) [file marinedrugs-10-01711-s001.pdf]
